# Supplementary figures and images for: Identification of novel plasma proteomic biomarkers of Dupuytren disease
Source: PLoS One. 2026 Mar 18;21(3):e0343733. doi: 10.1371/journal.pone.0343733 (PMC12998848; doi:10.1371/journal.pone.0343733)

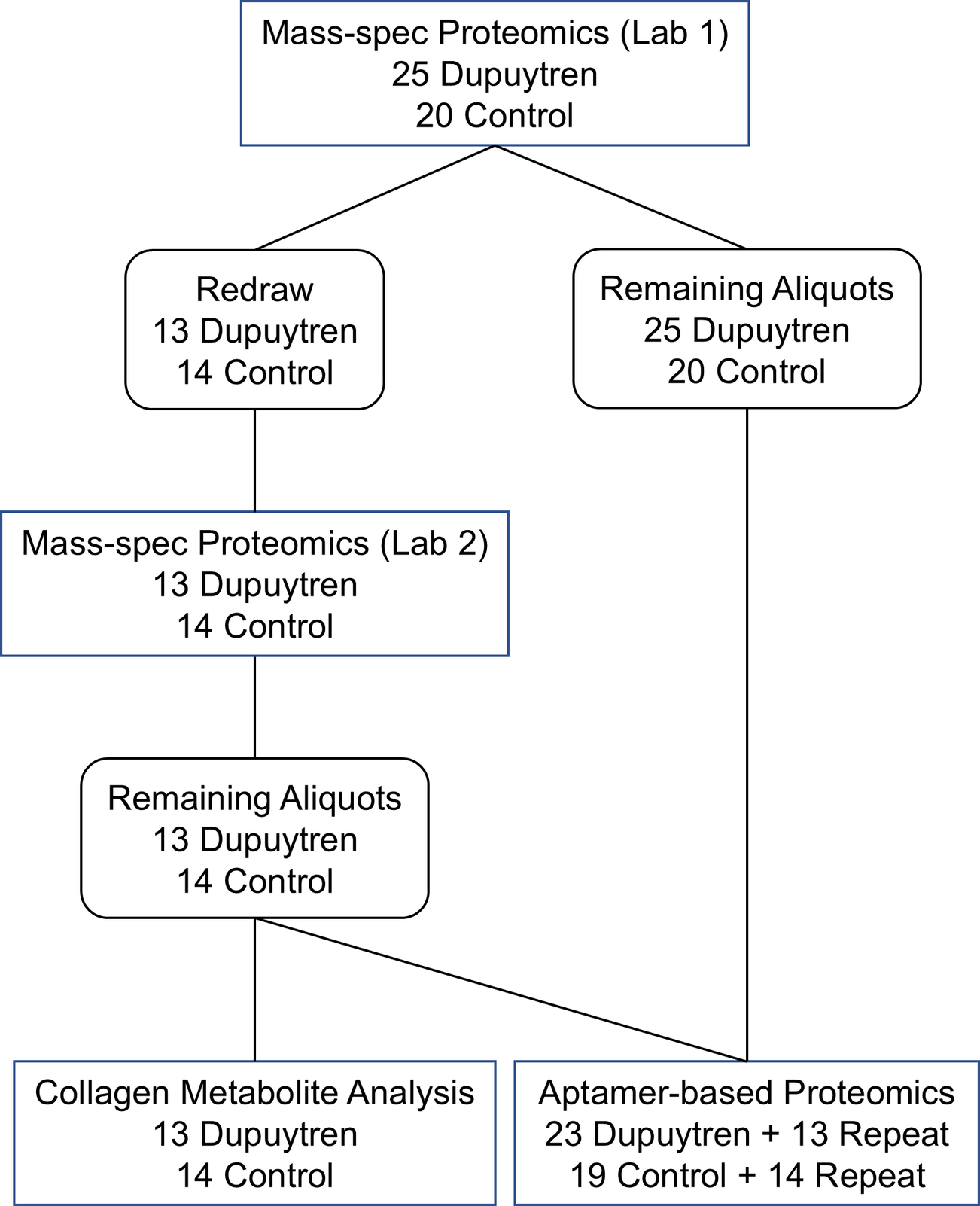

Supplement: S1 Fig — Forty-five subjects provided initial samples. Seventeen of these subjects had second samples drawn at least 6 months after the first. Mass spec lab 1 performed analysis on the initial 45 samples. Mass spec lab 2 and collagen metabolite analyses were performed on redraw specimens. Aptamer-based analysis was performed on both initial and redraw specimens. Three of the original forty-five samples for Aptamer analysis failed quality control, leaving 23 DD and 19 Controls available for analysis. (TIF) [file pone.0343733.s001.tif]

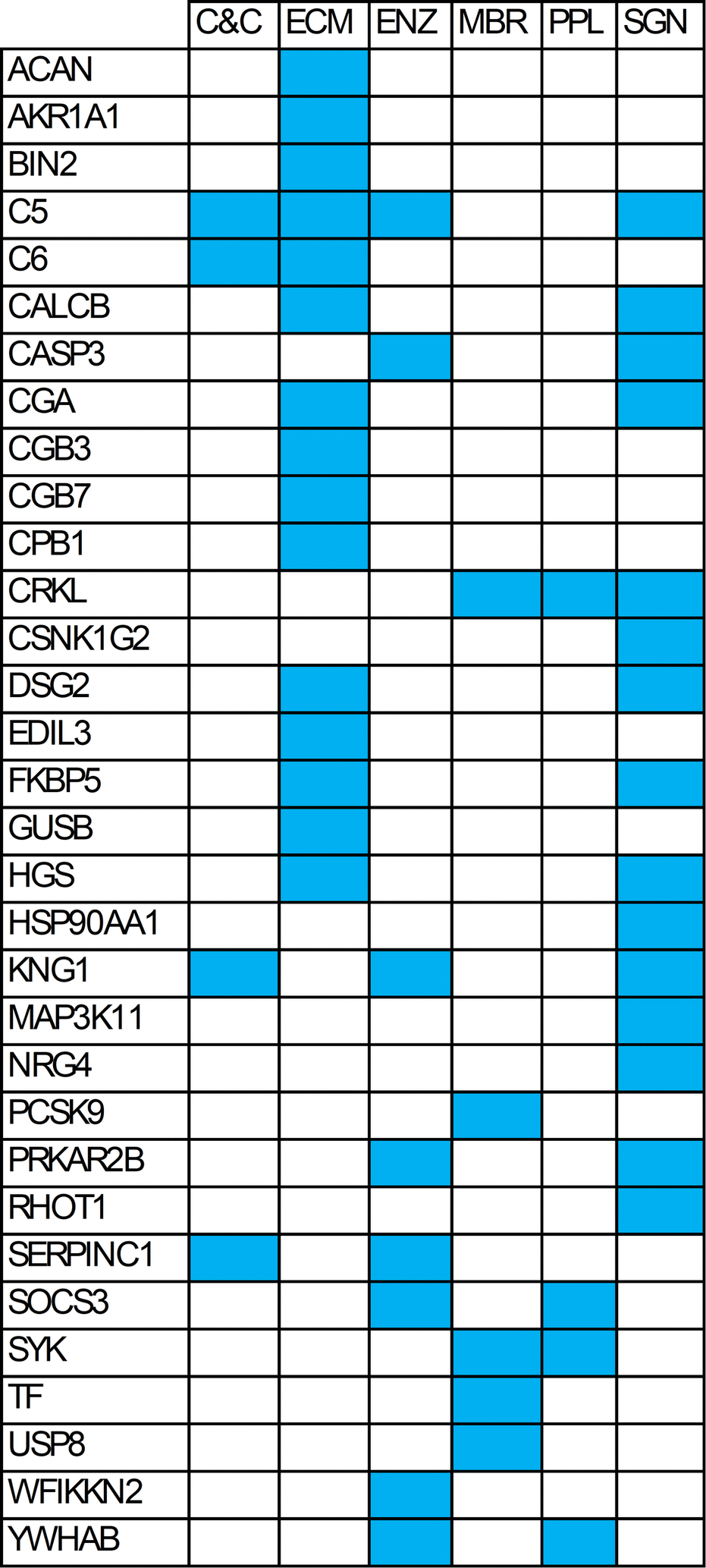

Supplement: S2 Fig — Thirty-two differentially expressed proteins showed significant network enrichment (p.adj < 0.05) in the following biological categories. C&C: coagulation and complement cascades, ECM: extracellular matrix, ENZ: Enzyme inhibition, MBR: cell membrane structures, PPL: phosphorylation, SGN: other signaling pathways. (TIF) [file pone.0343733.s002.tif]

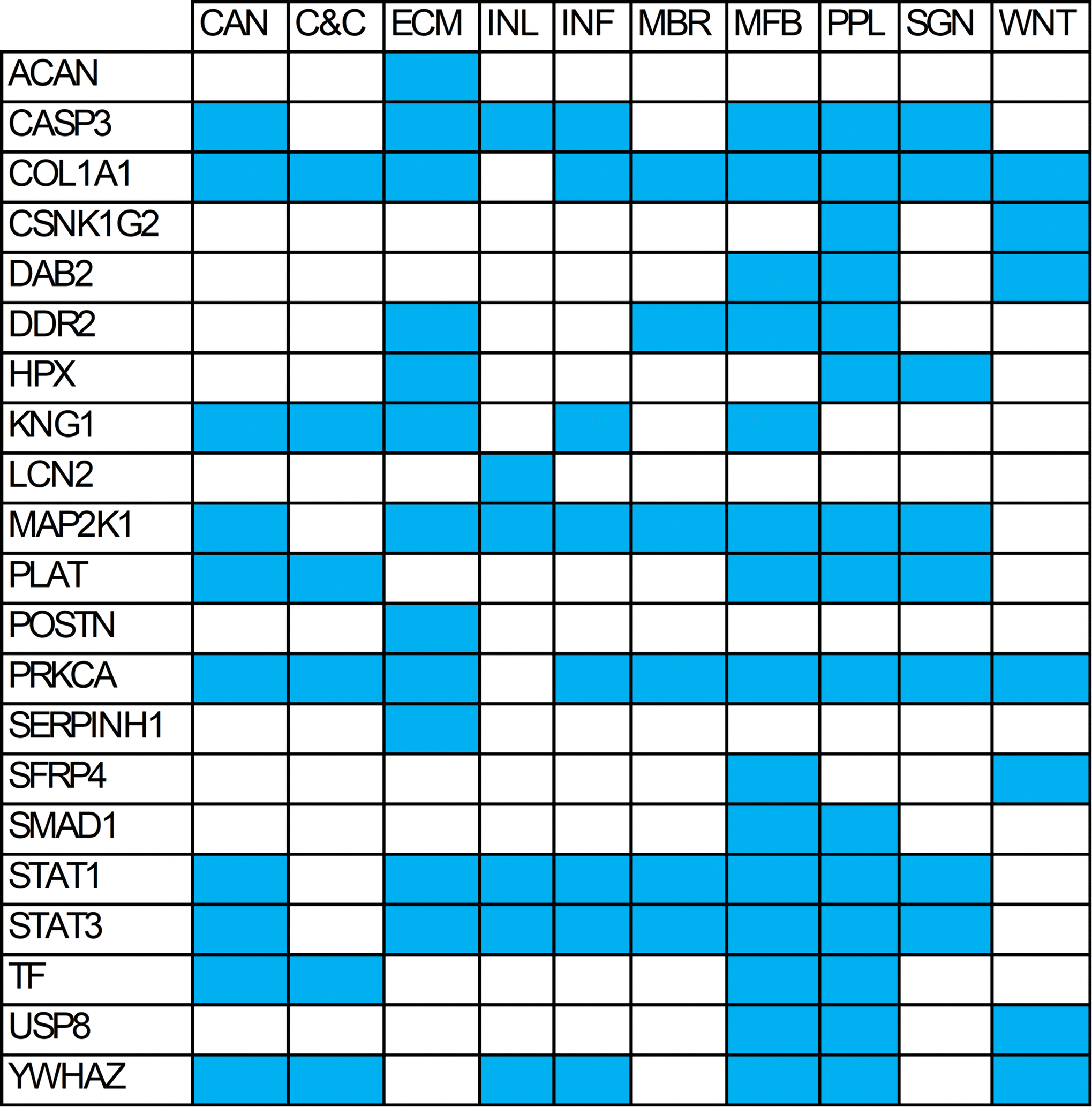

Supplement: S3 Fig — Twenty-one differentially expressed proteins showed significant network enrichment (p.adj < 0.05) in the following biological categories. CAN: Cancer, C&C: coagulation and complement cascades, ECM: extracellular matrix, INL: interleukin interactions, INF: infection, MBR: cell membrane structures, MFB: myofibroblast differentiation, proliferation, and motility, PPL: phosphorylation, SGN: other signaling pathways, WNT: WNT pathways. Hypothesis-based selection bias from a preselected list of candidate proteins may influence the enrichment results. (TIF) [file pone.0343733.s003.tif]

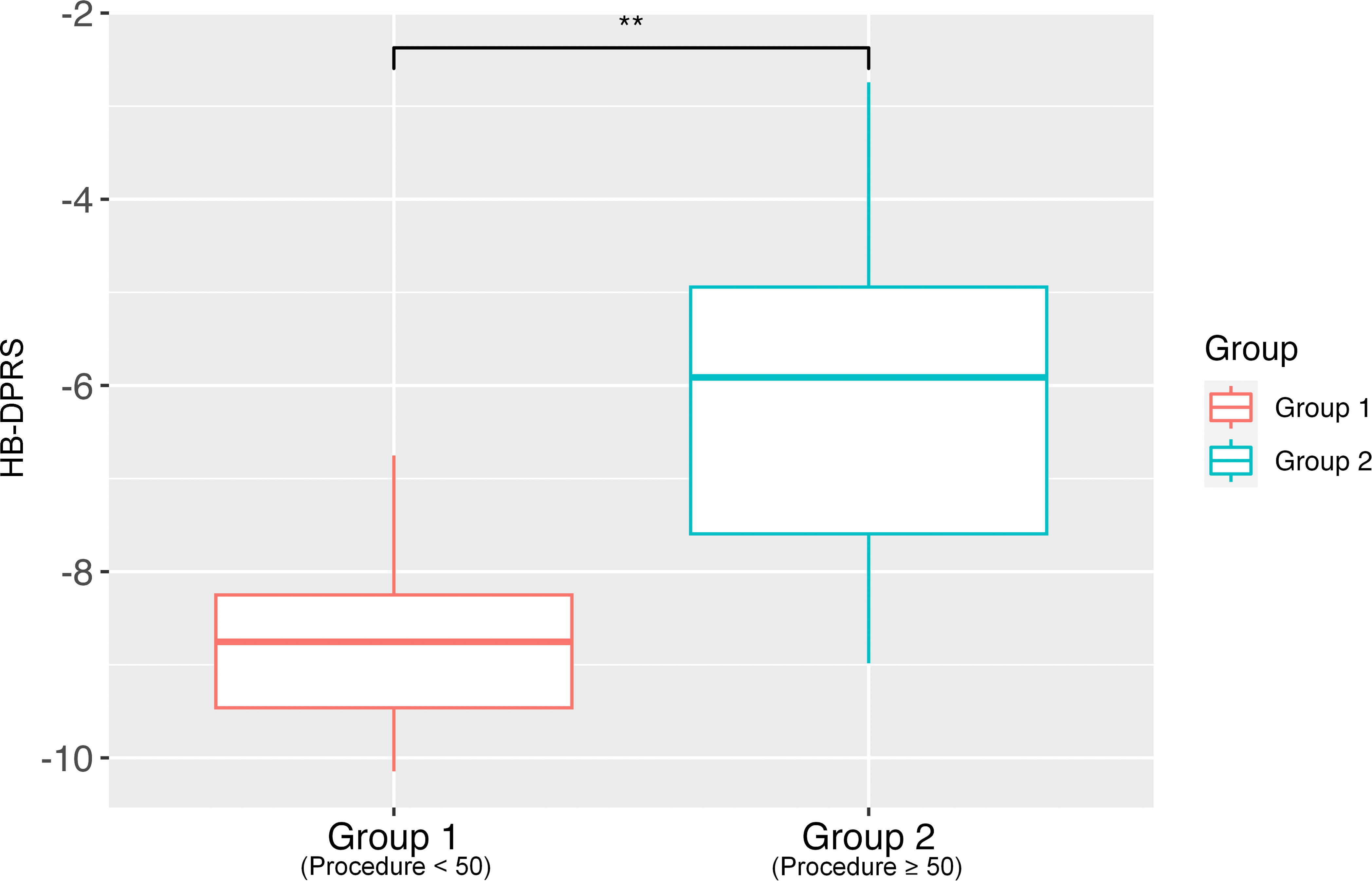

Supplement: S4 Fig — This 11-protein Hypothesis-Based Dupuytren Proteomic Risk Score (HB-DPRS) distinguished DD subjects with different disease progression rates based on whether the subject was younger than 50 vs. 50 or older during their first corrective procedure (p = 0.0018). (TIF) [file pone.0343733.s004.tif]

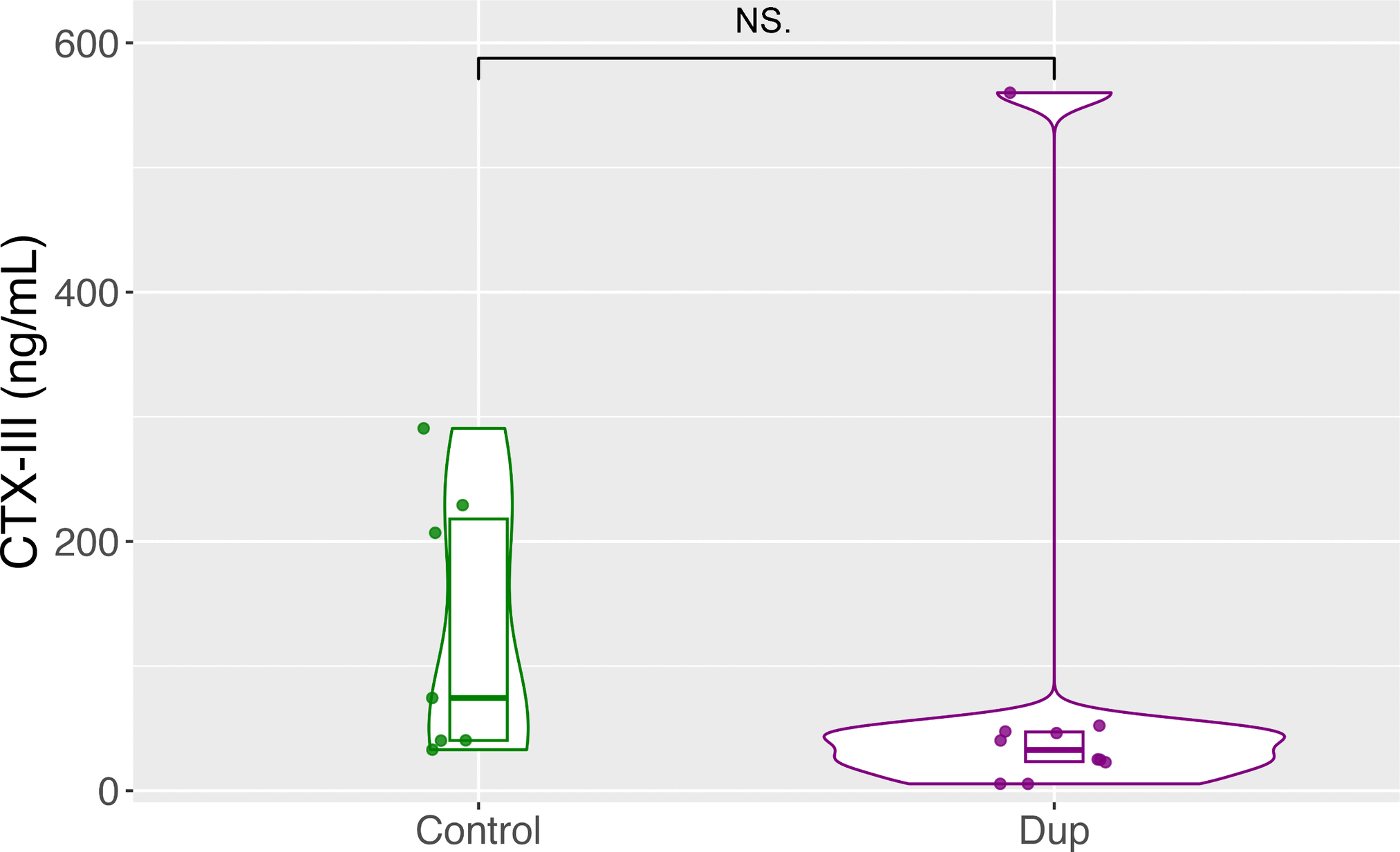

Supplement: S5 Fig — Although these violin plots visually suggest reduced Collagen III degradation marker CTX-III values in DD compared to controls, this difference was not significant (p-value = 0.4887). These data were from 17 of the 27 samples used for the other collagen metabolism markers, due to insufficient volumes in 10 samples. Because small cohorts magnify the effects of outliers, we recommend repeating these assays on larger cohorts. (TIF) [file pone.0343733.s005.tif]
