# Supplementary material for: Identification of novel plasma proteomic biomarkers of Dupuytren disease
Source: PLoS One. 2026 Mar 18;21(3):e0343733. doi: 10.1371/journal.pone.0343733 (PMC12998848; doi:10.1371/journal.pone.0343733)
Supplement: S1 Table — Of the 6995 aptamers in the Hypothesis-free analysis, 54 proteins showed statistically significant differences between the DD and control groups, with 24 overexpressed and 30 underexpressed after adjusting for multiple comparisons. Nine of these proteins were identified in both hypothesis-free and hypothesis-based analyses, as indicated by *. Plain italics indicate an indirect DD relationship, if any, to the protein; bold italics indicate a direct relationship between published DD findings and the protein. In the Notes column, “Referred to” means the referenced publication referred to the gene by an alias name. Exp: DD expression compared to controls. p-val: p-value of expression difference. FDR: False discovery rate (adjusted p-value. Nodes: number of pathway analysis connections (nodes) of this protein to others in this group with a medium confidence interaction score (0.400) and an FDR<=0.05. Notes: potential relationship to DD biology. Cat: categories of possible relationships to DD: 1. Apoptosis and senescence (3 genes); 2. Bioinformatics (9 genes); 3. Clinical and demographic (6 genes); 4. Extracellular matrix (6 genes); 5. Fibroblast and myofibroblast cytoskeleton, membrane, and motility (10 genes); 6. Fibroblast and myofibroblast differentiation and transcriptome (7 genes); 7. Vascular and perivascular (9 genes); Unclear relationship (16 genes). (DOCX) [file pone.0343733.s006.docx]

| **Gene** | **Express** | **p-val** | **FDR** | **Nodes** | **Notes** | **Cat** |
| --- | --- | --- | --- | --- | --- | --- |
| *ACAN** | Up | 0.001148 | 0.218 | 2 | Aggrecan core protein 2; Proteoglycan, binds to hyaluronic acid. ***ACAN is near DD-related SNP rs6496519 and is dysregulated in DD transcriptomic profiling*.** *Extracellular matrix interactions are key components of DD biology.* [1, 2, 3] | 2, 4 |
| *AKR1A1* | Down | 0.001021 | 0.218 | 1 | Aldo-keto reductase family 1 member A1. *A potential DD relationship is unclear*. | ? |
| *AOC3** | Up | 0.000845 | 0.218 | 0 | Membrane primary amine oxidase; cell adhesion protein. **Increased *AOC3 levels in small vessel endothelium cells in DD-affected tissues*.** (referred to by alias *VAP-1*), *DD is associated with local microvascular inflammation, thrombosis, and endothelial leucocyte adhesion.* [4, 5] | 7 |
| *ARG2* | Up | 0.000281 | 0.211 | 0 | Arginase-2, mitochondrial; promotes endothelial senescence, monocyte adhesion, and enhances *VCAM1 ⁄ ICAM1* levels. *DD is associated with local microvascular inflammation, thrombosis, and endothelial leucocyte adhesion*. [4, 6] | 7 |
| *BIN2* | Down | 0.001793 | 0.246 | 0 | Bridging integrator 2; Promotes cell motility and migration; central regulator of platelet activation in thrombosis and thrombo-inflammatory disease settings. *Cell-matrix, cell-cell, and cytoskeletal interactions are core processes in DD. DD is associated with local microvascular inflammation, thrombosis, and endothelial leucocyte adhesion.* [3, 4, 7] | 5, 7 |
| *C5* | Down | 0.001610 | 0.225 | 3 | Complement C5 alpha chain; initiates complement components, C5-C9 resulting in the Terminal Complement Complex, which is increased in the serum of Idiopathic Pulmonary Fibrosis; activates basophil and mast cell degranulation responsible for urticaria. *Itching is common in DD-affected tissues.* [8, 9, 10] | 3 |
| *C6* | Down | 0.001610 | 0.225 | 1 | Complement component C6. Forms part of the Terminal Complement Complex which is increased in the serum of Idiopathic Pulmonary Fibrosis. Upregulated in healing tendons subjected to mechanical loading; *DD fibroblast gene expression responds to mechanical loading.* [10, 11, 12] | 5 |
| *CALCB* | Up | 0.000333 | 0.211 | 1 | Calcitonin gene-related peptide 2. *A potential DD relationship is unclear.* | ? |
| *CASP3** | Down | 0.001603 | 0.075 | 8 | Caspase-3 subunit p12; Involved in apoptosis. ***PPI Network analysis projects key involvement in DD***. *Apoptosis is dysregulated in DD*. ***CASP3 is dysregulated in DD transcriptomic profiling***. [1, 13, 14] | 1, 2 |
| *CGA* | Up | 0.001093 | 0.218 | 2 | Glycoprotein hormones alpha chain; expression increases with age; *DD prevalence increases with age*. ***CGA is dysregulated in DD transcriptomic profiling***. [1, 8, 15] | 3 |
| *CGB3* | Up | 0.001093 | 0.218 | 1 | Chorionic gonadotropin subunit beta 3. *A potential DD relationship is unclear*. | ? |
| *CGB7* | Up | 0.001093 | 0.218 | 0 | Choriogonadotropin subunit beta 7. *A potential DD relationship is unclear*. | ? |
| *CPB1* | Up | 0.001398 | 0.222 | 2 | Carboxypeptidase B1. *A potential DD relationship is unclear*. | ? |
| *CRKL* | Down | 0.000892 | 0.218 | 3 | Crk-like protein; needed for fibroblast cytoskeletal structure and motility; epigenetic regulation of fibroblast focal adhesions. *Cell-matrix, cell-cell, and cytoskeletal interactions are core processes in DD*. [3, 16, 17] | 5 |
| *CSNK1G2** | Down | 0.001222 | 0.218 | 0 | Casein kinase I isoform gamma-2; Serine/threonine-protein kinase. Participates in WNT signaling. *WNT expression is dysregulated in DD; dysregulated in DD transcriptomic profiling.* ***Pathway analysis projects differential CSNK1G2 expression in both visibly affected and normal-appearing tissues in DD vs. control***. [1, 18, 19] | 2 |
| *DDX19A* | Down | 0.000886 | 0.218 | 0 | ATP-dependent RNA helicase DDX19A. *A potential DD relationship is unclear*. | ? |
| *DSG2* | Up | 0.001607 | 0.225 | 1 | Desmoglein-2; Fundamental component of intercellular desmosome junctions and cell-cell adhesion; involved in mechanosensitive and mechanoresponsive pathways. *Cell-matrix, cell-cell, and cytoskeletal interactions are core processes in DD*. [3, 20, 21] | 5 |
| *EDIL3* | Up | 0.000921 | 0.218 | 0 | EGF-like repeat and discoidin I-like domain-containing protein 3; Promotes adhesion of endothelial cells through interaction with the alpha-v/beta-3 integrin receptor. *DD is associated with local microvascular inflammation, thrombosis, and endothelial leucocyte adhesion*. [4, 22] | 7 |
| *EIF4H* | Down | 0.001031 | 0.218 | 1 | Eukaryotic translation initiation factor 4H; Stimulates the RNA helicase activity of EIF4A in the translation initiation complex. *EIF4A and ELN genes are in close proximity, and elastin loss is characteristic of DD-affected tissues*. [23, 24] | 4 |
| *ELAPOR1* | Up | 0.000609 | 0.218 | 0 | Endosome/Lysosome-Associated Apoptosis And Autophagy Regulator 1; involved in apoptosis and cell proliferation; may protect cells from cell death (referred to by alias EIG121). *Apoptosis is dysregulated in DD*. [14, 25, 26] | 1 |
| *FAM234B* | Up | 0.001227 | 0.218 | 2 | Protein FAM234B; predicted to be located in Golgi apparatus, cytoskeleton, and cell membrane. *Cell-matrix, cell-cell, and cytoskeletal interactions are core processes in DD*. [3] | 5 |
| *FKBP5* | Down | 0.001045 | 0.218 | 1 | Peptidyl-prolyl cis-trans isomerase FKBP5; involved in procollagen I triple helix assembly; *local collagen I accumulation is a prominent feature of DD. R*esponsible for the effects of mechanical loading on *COL1A1* and *COL1A2* expression (referred to by alias *PPIASE*); *DD fibroblast gene expression responds to mechanical loading*. [11, 27] | 4, 6 |
| *G3BP2* | Down | 0.001539 | 0.225 | 3 | Ras GTPase-activating protein-binding protein 2; binding varies with extracellular matrix stiffness; expression increased by mechanical stress; involved in endothelial shear stress-induced loss of endothelial barrier function, monocyte adhesion to endothelial cells, and proinflammatory cytokines. *DD is associated with local microvascular inflammation, thrombosis, and endothelial leucocyte adhesion. Cell-matrix, cell-cell, and cytoskeletal interactions are core processes in DD*. [3, 4, 28, 29] | 5, 7 |
| *GP1BB* | Down | 0.000767 | 0.218 | 1 | Platelet glycoprotein Ib beta chain; transmembrane platelet protein; binds to subendothelial von Willebrand factor; involved in forming platelet plugs; necessary for normal platelet adhesion to a stimulated endothelium. *DD is associated with local microvascular inflammation, thrombosis, and endothelial leucocyte adhesion*. [4, 30] | 7 |
| *GUSB* | Down | 0.000113 | 0.185 | 1 | Beta-glucuronidase; degrades extracellular matrix glycosaminoglycans, including heparan sulfate, dermatan sulfate, and chondroitin-4, 6-sulfate. *Extracellular matrix remodeling is a prominent process in DD*. [3, 31] | 5 |
| *HGS* | Down | 0.000178 | 0.208 | 7 | Hepatocyte growth factor-regulated tyrosine kinase substrate. *A potential DD relationship is unclear*. | ? |
| *HSP90AA1* | Down | 0.001139 | 0.218 | 0 | Heat shock protein HSP 90-alpha; activates endothelial (KNG1) bradykinin-forming cascade; activates MMP2; in combination, TGF-β1 and Hsp90β stimulate anchorage-independent growth, reduce adhesion and stimulate migration via an alternate TGF-β1 pathway, mediated by αvβ6 integrin. *Cell-matrix, cell-cell, and cytoskeletal interactions are core processes in DD*. [3, 32, 33] | 4, 5 |
| *KNG1** | Up | 0.000490 | 0.218 | 6 | Kininogen1; Alternative splicing produces high molecular weight kininogen (HMWK) and low molecular weight kininogen (LMWK). HMWK inhibits thrombin- and plasmin-induced thrombocyte aggregation; stimulates the release of other mediators of inflammation; causes vasodilation and increases vascular permeability; the bradykinin B2 receptor contributes to endothelial inflammation. *DD is associated with local microvascular inflammation, thrombosis, and endothelial leucocyte adhesion*. [4, 34] | 7 |
| *MAP3K11* | Up | 0.001532 | 0.225 | 2 | Mitogen-activated protein kinase kinase kinase 11; plays a role in the activation of BRAF. *BRAF inhibitors provoke DD-like clinical changes*. [35] | 3 |
| *MBNL1* | Down | 0.001278 | 0.218 | 1 | Muscleblind-like protein 1; central regulator of fibrosis; regulates αSMA; regulates the TGF-β/Smad signaling pathway; *SMAD - TGF are prominent pathways in DD fibroblast proliferation and contraction*. [36, 37] | 6 |
| *MBNL2* | Down | 0.000971 | 0.218 | 1 | Muscleblind-like protein 2; Mediates pre-mRNA alternative splicing regulation; part of an RNA-binding protein network governing fibroblast to myofibroblast transition. *Fibroblast differentiation into myofibroblast phenotype is a core process in DD*. [38, 39] | 6 |
| *NAA80* | Down | 0.001251 | 0.218 | 0 | N-alpha-acetyltransferase 80; regulates actin filament acetylation, depolymerization, and elongation; plays essential roles in filament assembly, cytoskeleton organization, and cell motility. *Cell-matrix, cell-cell, and cytoskeletal interactions are core processes in DD*. [3, 40] | 5 |
| *NRG4* | Up | 0.000669 | 0.218 | 1 | Pro-neuregulin-4, membrane-bound isoform; brown fat adipokine; lower levels in obese compared to normal-weight. *DD is associated with loss of subcutaneous palm fat and lower triceps skinfold thickness compared with normal*. [41-43] | 3 |
| *OSCAR* | Up | 0.000070 | 0.185 | 1 | Osteoclast-associated immunoglobulin-like receptor; collagen I is an *OSCAR* ligand. *OSCAR* also stimulates the release of TNFα from T-cells. *TNF is involved in DD pathways*. [44, 45] | 2 |
| *PCSK9* | Up | 0.001371 | 0.222 | 1 | Proprotein convertase subtilisin/kexin type 9; blocks LDL degradation and increases circulating LDL. *DD is associated with increased mortality from cardiovascular disease*. [46] | 3 |
| *POSTN** | Up | 0.000312 | 0.211 | 2 | Periostin; Induces cell attachment and spreading and plays a role in cell adhesion. Enhances incorporation of BMP1 in the fibronectin matrix of connective tissues, and subsequent proteolytic activation of lysyl oxidase LOX. *Increased LOX activity in Dupuytren tissue.* ***Increased POSTN expression in DD fibroblasts and in sweat glands of DD-adjacent skin****.* [1, 20, 47-49] | 4, 6 |
| *PRKAR2B* | Down | 0.001119 | 0.218 | 0 | cAMP-dependent protein kinase type II-beta regulatory subunit. *A potential DD relationship is unclear*. | ? |
| *PRSS1* | Up | 0.000809 | 0.218 | 1 | Alpha-trypsin chain 1; Belongs to the peptidase S1 family; expressed in the pancreas and some malignancies. Activates several metalloproteinases, but only where locally expressed. *A potential DD relationship is unclear*. | ? |
| *RAB24* | Down | 0.000235 | 0.211 | 0 | Ras-related protein Rab-24; involved in autophagy-related processes; overexpressed in senescent fibroblasts. *DD fibroblasts have altered senescence pathways*. [50] | 1 |
| *RHOT1* | Down | 0.000133 | 0.185 | 0 | Mitochondrial Rho GTPase 1; essential for dynamic positioning of mitochondria along the length of axons; mitochondrial positioning along the length of axons and trafficking; impaired function is associated with neurodegenerative disease; (referred to by alias MIRO). *Progressive Pacinian corpuscle denervation is associated with DD*. [51, 52] | 3 |
| *SCGN* | Up | 0.000085 | 0.185 | 0 | Secretagogin, cytoplasmic calcium binding protein. *A potential DD relationship is unclear*. | ? |
| *SCPEP1* | Down | 0.000439 | 0.218 | 0 | Retinoid-inducible serine carboxypeptidase*. A potential DD relationship is unclear*. | ? |
| *SERPINC1* | Up | 0.001500 | 0.225 | 4 | Antithrombin-III; regulates the blood coagulation cascade; inhibits thrombin, and factors IXa, Xa and Xia; binds to endothelial heparin-like molecules. Deficiency or loss-of-function variants are major risk factors for thromboembolic disease. (referred to by alias Antithrombin III). *DD is associated with local microvascular inflammation, thrombosis, and endothelial leucocyte adhesion*. [4] | 7 |
| *SHMT1* | Down | 0.000619 | 0.218 | 0 | Serine hydroxymethyltransferase, cytosolic; Interconversion of serine and glycine. Nearly one third of collagen fibril composition is glycine, but the *potential impact of SHMT1 on DD-related collagen accumulation is unclear*. | ? |
| *SMAD1** | Down | 0.001275 | 0.218 | 0 | Mothers against decapentaplegic homolog 1; Transcriptional modulator activated by BMP (bone morphogenetic proteins) type 1 receptor kinase. SMAD1/5/9 pathway action is antifibrotic in multiple organ fibroses. *SMAD-TGFβ pathways are prominent in DD*. ***SMAD1 protein expression is normal in DD tissues, but intracellular SMAD1 mRNA expression is reduced in DD fibroblasts*.** [36] | 2, 6 |
| *SOCS3* | Down | 0.001007 | 0.218 | 3 | Suppressor of cytokine signaling 3. SOCS3 is involved in negative regulation of cytokines that signal through the JAK/STAT pathway. *Feedback loop regulation of DD-related cytokines, including IL6 and TNF*. *DD-related fibrosis involves JAK/STAT/IL13 signaling pathways*. [53] | 2 |
| *SPART* | Down | 0.000247 | 0.211 | 0 | Spartin; Participates in cytokinesis. SPART is associated with Epidermal Growth Factor Receptor (EGFR) degradation and transport to the cell membrane; *an increased ratio of intracellular to cell membrane EGFR has been found in early DD disease and is upregulated in cell culture.* ***SPART is under-expressed in DD Fibroblasts by Genome-wide exon expression profiles*** (referred to by alias SPG20). [54, 55] | 2, 6 |
| *SPATA22* | Up | 0.000871 | 0.218 | 0 | Spermatogenesis-associated protein 22. *A potential DD relationship is unclear*. | ? |
| *SYK* | Down | 0.000513 | 0.218 | 6 | Tyrosine-protein kinase SYK; regulates focal adhesion kinase. Integrins and related cell membrane adhesion and mechanosensing domains. *Cell-matrix, cell-cell, and cytoskeletal interactions are core processes in DD*. [3, 56] | 5 |
| *TBC1D13* | Down | 0.001076 | 0.218 | 0 | TBC1 domain family member 13. *A potential DD relationship is unclear*. | ? |
| *TF** | Up | 0.000025 | 0.172 | 2 | Serotransferrin; Responsible for the transport of iron from sites of absorption and heme degradation; iron levels are increased in the lung tissues of patients with idiopathic pulmonary fibrosis; exogenous iron increases human lung fibroblast proliferation and cytokine responses. *Local tissue iron deposition occurs in the early cellular stage of DD*. [57, 58] | 4, 7 |
| *USP8** | Down | 0.001398 | 0.222 | 2 | Ubiquitin carboxyl-terminal hydrolase 8; Hydrolase that can remove conjugated ubiquitin; prevents Wnt receptor frizzled 5 (FZD5); USP8 pathway is essential for Wnt/β-catenin signaling; *WNT signaling pathways are central in DD biology*. ***USP8 is under-expressed in DD Fibroblasts by Genome-wide exon expression profiles***. USP8 may exert protective influences against aging; *DD prevalence is age-related*. [54, 59] | 2, 3 |
| *WFIKKN2* | Up | 0.001128 | 0.218 | 0 | WAP, Kazal, immunoglobulin, Kunitz, and NTR domain-containing protein 2; Probably has serine protease- and metalloprotease-inhibitor activity. *MMP inhibition plays a role in DD biology*. Regulates the balance between the activation of Smad and non-Smad pathways by TGFB1. *SMAD-TGFβ pathways are prominent in DD.* [36] | 2, 6 |
| *YWHAB* | Down | 0.001257 | 0.218 | 4 | 14-3-3 protein beta/alpha, N-terminally processed. *A potential DD relationship is unclear*. | ? |

**S1 Table. 54 Differentially expressed genes in the SomaScan Hypothesis-free analysis**. Of the 6995 aptamers in the Hypothesis-free analysis, 54 proteins showed statistically significant differences between the DD and control groups, with 24 overexpressed and 30 underexpressed after adjusting for multiple comparisons. Nine of these proteins were identified in both hypothesis-free and hypothesis-based analyses, as indicated by *. *Plain italics* indicate an indirect DD relationship, if any, to the protein; ***bold italics*** indicate a direct relationship between published DD findings and the protein. In the Notes column, "Referred to" means the referenced publication referred to the gene by an alias name. Exp: DD expression compared to controls. p-val: p-value of expression difference. FDR: False discovery rate (adjusted p-value. Nodes: number of pathway analysis connections (nodes) of this protein to others in this group with a medium confidence interaction score (0.400) and an FDR<=0.05. Notes: potential relationship to DD biology. Cat: categories of possible relationships to DD: 1. Apoptosis and senescence (3 genes); 2. Bioinformatics (9 genes); 3. Clinical and demographic (6 genes); 4. Extracellular matrix (6 genes); 5. Fibroblast and myofibroblast cytoskeleton, membrane, and motility (10 genes); 6. Fibroblast and myofibroblast differentiation and transcriptome (7 genes); 7. Vascular and perivascular (9 genes); Unclear relationship (16 genes).

**S1 Table References**

1. Rehman S, Salway F, Stanley JK, Ollier WE, Day P, Bayat A. Molecular phenotypic descriptors of Dupuytren's disease defined using informatics analysis of the transcriptome. J Hand Surg Am. 2008;33(3):359–72. Epub 2008/03/18. doi: 10.1016/j.jhsa.2007.11.010. PubMed PMID: 18343292.

2. Ng M, Thakkar D, Southam L, Werker P, Ophoff R, Becker K, et al. A Genome-wide Association Study of Dupuytren Disease Reveals 17 Additional Variants Implicated in Fibrosis. Am J Hum Genet. 2017;101(3):417–27. Epub 2017/09/09. doi: 10.1016/j.ajhg.2017.08.006. PubMed PMID: 28886342; PubMed Central PMCID: PMC5591021.

3. O’Gorman DB. The Extracellular Matrix in Dupuytren Disease. Dupuytren Disease and Related Diseases - The Cutting Edge 2017. p. 43–54.

4. Mayerl C, Del Frari B, Parson W, Boeck G, Piza-Katzer H, Wick G, et al. Characterisation of the inflammatory response in Dupuytren's disease. J Plast Surg Hand Surg. 2016;50(3):171–9. Epub 2016/02/09. doi: 10.3109/2000656X.2016.1140054. PubMed PMID: 26852784.

5. Pannecoeck R, Serruys D, Benmeridja L, Delanghe JR, van Geel N, Speeckaert R, et al. Vascular adhesion protein-1: Role in human pathology and application as a biomarker. Crit Rev Clin Lab Sci. 2015;52(6):284–300. Epub 20150818. doi: 10.3109/10408363.2015.1050714. PubMed PMID: 26287391.

6. Yepuri G, Velagapudi S, Xiong Y, Rajapakse AG, Montani JP, Ming XF, et al. Positive crosstalk between arginase-II and S6K1 in vascular endothelial inflammation and aging. Aging Cell. 2012;11(6):1005–16. Epub 20120918. doi: 10.1111/acel.12001. PubMed PMID: 22928666.

7. Volz J, Kusch C, Beck S, Popp M, Vogtle T, Meub M, et al. BIN2 orchestrates platelet calcium signaling in thrombosis and thrombo-inflammation. J Clin Invest. 2020;130(11):6064–79. doi: 10.1172/JCI136457. PubMed PMID: 32750041; PubMed Central PMCID: PMC7598067.

8. Eaton C. The Next Stage of Clinical Dupuytren Research: Biomarkers and Chronic Disease Research Tools. In: al. PMNWe, editor. Dupuytren Disease and Related Diseases - The Cutting Edge. Switzerland: Springer; 2017. p. 391–407.

9. Yanase Y, Matsuo Y, Takahagi S, Kawaguchi T, Uchida K, Ishii K, et al. Coagulation factors induce human skin mast cell and basophil degranulation via activation of complement 5 and the C5a receptor. J Allergy Clin Immunol. 2021;147(3):1101–4 e7. Epub 20200902. doi: 10.1016/j.jaci.2020.08.018. PubMed PMID: 32888945.

10. Sikkeland LIB, Ueland T, Lund MB, Durheim MT, Mollnes TE. A role for the terminal C5-C9 complement pathway in idiopathic pulmonary fibrosis. Front Med (Lausanne). 2023 Aug 9:10:1236495. doi: 10.3389/fmed.2023.1236495. eCollection 2023.

11. Verhoekx JS, Beckett KS, Bisson MA, McGrouther DA, Grobbelaar AO, Mudera V. The mechanical environment in Dupuytren's contracture determines cell contractility and associated MMP-mediated matrix remodeling. J Orthop Res. 2013;31(2):328–34. Epub 2012/09/19. doi: 10.1002/jor.22220. PubMed PMID: 22987740.

12. Hammerman M, Blomgran P, Dansac A, Eliasson P, Aspenberg P. Different gene response to mechanical loading during early and late phases of rat Achilles tendon healing. J Appl Physiol (1985). 2017;123(4):800–15. Epub 20170713. doi: 10.1152/japplphysiol.00323.2017. PubMed PMID: 28705996.

13. Stocks M, Walter AS, Akova E, Gauglitz G, Aszodi A, Boecker W, et al. RNA-seq unravels distinct expression profiles of keloids and Dupuytren's disease. Heliyon. 2024;10(1):e23681. Epub 20231213. doi: 10.1016/j.heliyon.2023.e23681. PubMed PMID: 38187218; PubMed Central PMCID: PMC10770622.

14. Wilutzky B, Berndt A, Katenkamp D, Koshmehl H. Programmed cell death in nodular palmar fibromatosis (Morbus Dupuytren). Histol Histopathol. 1998;13(1):67–72. Epub 1998/02/26. doi: 10.14670/HH-13.67. PubMed PMID: 9476635.

15. Tanaka T, Biancotto A, Moaddel R, Moore AZ, Gonzalez-Freire M, Aon MA, et al. Plasma proteomic signature of age in healthy humans. Aging Cell. 2018;17(5):e12799. Epub 2018/07/12. doi: 10.1111/acel.12799. PubMed PMID: 29992704; PubMed Central PMCID: PMC6156492.

16. Park TJ, Curran T. Essential roles of Crk and CrkL in fibroblast structure and motility. Oncogene. 2014;33(43):5121–32. Epub 20131028. doi: 10.1038/onc.2013.453. PubMed PMID: 24166500.

17. Kanazawa T, Michida H, Uchino Y, Ishihara A, Zhang S, Tabata S, et al. Cell shape-based chemical screening reveals an epigenetic network mediated by focal adhesions. FEBS J. 2021;288(19):5613–28. Epub 20210422. doi: 10.1111/febs.15840. PubMed PMID: 33768715.

18. van Beuge MM, Ten Dam EJ, Werker PM, Bank RA. Wnt pathway in Dupuytren disease: connecting profibrotic signals. Transl Res. 2015;166(6):762–71 e3. Epub 2015/10/17. doi: 10.1016/j.trsl.2015.09.006. PubMed PMID: 26470681.

19. Satish L, LaFramboise WA, Johnson S, Vi L, Njarlangattil A, Raykha C, et al. Fibroblasts from phenotypically normal palmar fascia exhibit molecular profiles highly similar to fibroblasts from active disease in Dupuytren's Contracture. BMC Med Genomics. 2012;5:15. Epub 2012/05/09. doi: 10.1186/1755-8794-5-15. PubMed PMID: 22559715; PubMed Central PMCID: PMC3375203.

20. Verhoekx JSN, Verjee LS, Izadi D, Chan JKK, Nicolaidou V, Davidson D, et al. Isometric contraction of Dupuytren's myofibroblasts is inhibited by blocking intercellular junctions. J Invest Dermatol. 2013;133(12):2664–71. Epub 2013/05/09. doi: 10.1038/jid.2013.219. PubMed PMID: 23652794.

21. Moch M, Schieren J, Leube RE. Cortical tension regulates desmosomal morphogenesis. Front Cell Dev Biol. 2022;10:946190. Epub 20221004. doi: 10.3389/fcell.2022.946190. PubMed PMID: 36268507; PubMed Central PMCID: PMC9577410.

22. Ryu YH, Lee YJ, Kim KJ, Lee SJ, Han YN, Rhie JW. Epidermal Growth Factor (EGF)-Like Repeats and Discoidin I-Like Domains 3 (EDIL3): A Potential New Therapeutic Tool for the Treatment of Keloid Scars. Tissue Eng Regen Med. 2017;14(3):267–77. Epub 20170407. doi: 10.1007/s13770-017-0034-5. PubMed PMID: 30603483; PubMed Central PMCID: PMC6171597.

23. Ferrero GB, Howald C, Micale L, Biamino E, Augello B, Fusco C, et al. An atypical 7q11.23 deletion in a normal IQ Williams-Beuren syndrome patient. Eur J Hum Genet. 2010;18(1):33–8. doi: 10.1038/ejhg.2009.108. PubMed PMID: 19568270; PubMed Central PMCID: PMC2987159.

24. Millesi H, Reihsner R, Hamilton G, Mallinger R, Menzel EJ. Biomechanical properties of normal tendons, normal palmar aponeuroses and palmar aponeuroses from patients with Dupuytren's disease subjected to elastase and chondroitinase treatment. Connect Tissue Res. 1995;31(2):109–15. doi: 10.3109/03008209509028398. PubMed PMID: 15612326.

25. Deng L, Feng J, Broaddus RR. The novel estrogen-induced gene EIG121 regulates autophagy and promotes cell survival under stress. Cell Death Dis. 2010;1(4):e32. doi: 10.1038/cddis.2010.9. PubMed PMID: 21072319; PubMed Central PMCID: PMC2976047.

26. Jemec B, Grobbelaar AO, Wilson GD, Smith PJ, Sanders R, McGrouther DA. Is Dupuytren's disease caused by an imbalance between proliferation and cell death? J Hand Surg Br. 1999;24(5):511–4. Epub 1999/12/22. doi: 10.1054/jhsb.1999.0251. PubMed PMID: 10597921.

27. Kaku M, Yamauchi M. Mechano-regulation of collagen biosynthesis in periodontal ligament. J Prosthodont Res. 2014;58(4):193–207. Epub 2014/10/15. doi: 10.1016/j.jpor.2014.08.003. PubMed PMID: 25311991; PubMed Central PMCID: PMC4253671.

28. Li T, Qiu J, Jia T, Liang Y, Zhang K, Yan W, et al. G3BP2 regulates oscillatory shear stress-induced endothelial dysfunction. Genes Dis. 2022;9(6):1701–15. Epub 20211119. doi: 10.1016/j.gendis.2021.11.003. PubMed PMID: 36157502; PubMed Central PMCID: PMC9485288.

29. Wei SC, Fattet L, Tsai JH, Guo Y, Pai VH, Majeski HE, et al. Matrix stiffness drives epithelial-mesenchymal transition and tumour metastasis through a TWIST1-G3BP2 mechanotransduction pathway. Nat Cell Biol. 2015;17(5):678–88. Epub 20150420. doi: 10.1038/ncb3157. PubMed PMID: 25893917; PubMed Central PMCID: PMC4452027.

30. Modjeski KL, Ture SK, Field DJ, Cameron SJ, Morrell CN. Glutamate Receptor Interacting Protein 1 Mediates Platelet Adhesion and Thrombus Formation. PLoS One. 2016;11(9):e0160638. Epub 20160915. doi: 10.1371/journal.pone.0160638. PubMed PMID: 27631377; PubMed Central PMCID: PMC5025166.

31. Tomatsu S, Montano AM, Dung VC, Grubb JH, Sly WS. Mutations and polymorphisms in GUSB gene in mucopolysaccharidosis VII (Sly Syndrome). Hum Mutat. 2009;30(4):511–9. doi: 10.1002/humu.20828. PubMed PMID: 19224584; PubMed Central PMCID: PMC3048808.

32. Chakraborty A, Edkins AL. HSP90 as a regulator of extracellular matrix dynamics. Biochem Soc Trans. 2021;49(6):2611–25. doi: 10.1042/BST20210374. PubMed PMID: 34913470.

33. de la Mare JA, Jurgens T, Edkins AL. Extracellular Hsp90 and TGFbeta regulate adhesion, migration and anchorage independent growth in a paired colon cancer cell line model. BMC Cancer. 2017;17(1):202. Epub 20170316. doi: 10.1186/s12885-017-3190-z. PubMed PMID: 28302086; PubMed Central PMCID: PMC5356307.

34. Terzuoli E, Corti F, Nannelli G, Giachetti A, Donnini S, Ziche M. Bradykinin B2 Receptor Contributes to Inflammatory Responses in Human Endothelial Cells by the Transactivation of the Fibroblast Growth Factor Receptor FGFR-1. Int J Mol Sci. 2018;19(9). Epub 20180906. doi: 10.3390/ijms19092638. PubMed PMID: 30200598; PubMed Central PMCID: PMC6163484.

35. Vandersleyen V, Grosber M, Wilgenhof S, De Kock J, Neyns B, Gutermuth J. Vemurafenib-associated Dupuytren- and Ledderhose palmoplantar fibromatosis in metastatic melanoma patients. J Eur Acad Dermatol Venereol. 2016;30(7):1133–5. Epub 20150824. doi: 10.1111/jdv.13268. PubMed PMID: 26303964.

36. Krause C, Kloen P, Ten Dijke P. Elevated transforming growth factor beta and mitogen-activated protein kinase pathways mediate fibrotic traits of Dupuytren's disease fibroblasts. Fibrogenesis Tissue Repair. 2011;4(1):14. Epub 2011/06/30. doi: 10.1186/1755-1536-4-14. PubMed PMID: 21711521; PubMed Central PMCID: PMC3148569.

37. Higuchi Y, Ogata T, Nakanishi N, Nishi M, Tsuji Y, Tomita S, et al. Cavin-2 promotes fibroblast-to-myofibroblast trans-differentiation and aggravates cardiac fibrosis. ESC Heart Fail. 2023. Epub 20231024. doi: 10.1002/ehf2.14571. PubMed PMID: 37872863.

38. Gokel JM, Hübner G. Occurrence of Myofibroblasts in the Different Phases of Morbus Dupuytren (Dupuytren's Contracture). Beiträge zur Pathologie. 1977;161(2):166–75. doi: 10.1016/s0005-8165(77)80095-4.

39. Chothani S, Schafer S, Adami E, Viswanathan S, Widjaja AA, Langley SR, et al. Widespread Translational Control of Fibrosis in the Human Heart by RNA-Binding Proteins. Circulation. 2019;140(11):937–51. Epub 20190709. doi: 10.1161/CIRCULATIONAHA.119.039596. PubMed PMID: 31284728; PubMed Central PMCID: PMC6749977.

40. Drazic A, Aksnes H, Marie M, Boczkowska M, Varland S, Timmerman E, et al. NAA80 is actin's N-terminal acetyltransferase and regulates cytoskeleton assembly and cell motility. Proc Natl Acad Sci U S A. 2018;115(17):4399–404. Epub 20180326. doi: 10.1073/pnas.1718336115. PubMed PMID: 29581253; PubMed Central PMCID: PMC5924898.

41. Bergenudd H, LindgÄRde F, Nilsson BE. Prevalence of Dupuytren’s Contracture and its Correlation with Degenerative Changes of the Hands and Feet and with Criteria of General Health. Journal of Hand Surgery. 2016;18(2):254–7. doi: 10.1016/0266-7681(93)90123-w.

42. Flint MH. The genesis of the palmar lesion. In: McFarlane RM, McGrouther DA, Flint MH, editors. Dupuytren's Disease Biology and Treatment. Edinburg Churchill Livingstone; 1990.

43. Worku MG, Seretew WS, Angaw DA, Tesema GA. Prevalence and Associated Factor of Brown Adipose Tissue: Systematic Review and Meta-Analysis. Biomed Res Int. 2020;2020:9106976. Epub 20200616. doi: 10.1155/2020/9106976. PubMed PMID: 32685543; PubMed Central PMCID: PMC7317326.

44. Verjee LS, Verhoekx JS, Chan JK, Krausgruber T, Nicolaidou V, Izadi D, et al. Unraveling the signaling pathways promoting fibrosis in Dupuytren's disease reveals TNF as a therapeutic target. Proc Natl Acad Sci U S A. 2013;110(10):E928–37. Epub 2013/02/23. doi: 10.1073/pnas.1301100110. PubMed PMID: 23431165; PubMed Central PMCID: PMC3593900.

45. Schultz HS, Nitze LM, Zeuthen LH, Keller P, Gruhler A, Pass J, et al. Collagen induces maturation of human monocyte-derived dendritic cells by signaling through osteoclast-associated receptor. J Immunol. 2015;194(7):3169–79. Epub 20150227. doi: 10.4049/jimmunol.1402800. PubMed PMID: 25725106; PubMed Central PMCID: PMC4367160.

46. Kuo RYL, Ng M, Prieto-Alhambra D, Furniss D. Dupuytren's Disease Predicts Increased All-Cause and Cancer-Specific Mortality: Analysis of a Large Cohort from the U.K. Clinical Practice Research Datalink. Plast Reconstr Surg. 2020;145(3):574e–82e. Epub 2020/02/26. doi: 10.1097/PRS.0000000000006551. PubMed PMID: 32097318; PubMed Central PMCID: PMC7043723.

47. Hamamoto H, Ueba Y, Sudo Y, Sanada H, Yamamuro T, Takeda T. Dupuytren's contracture: morphological and biochemical changes in palmar aponeurosis. Hand. 1982;14(3):237–47. Epub 1982/10/01. doi: 10.1016/s0072-968x(82)80056-9. PubMed PMID: 6130030.

48. Shih B, Brown JJ, Armstrong DJ, Lindau T, Bayat A. Differential gene expression analysis of subcutaneous fat, fascia, and skin overlying a Dupuytren's disease nodule in comparison to control tissue. Hand (N Y). 2009;4(3):294–301. Epub 2009/02/03. doi: 10.1007/s11552-009-9164-0. PubMed PMID: 19184239; PubMed Central PMCID: PMC2724615.

49. Tripkovic I, Ogorevc M, Vukovic D, Saraga-Babic M, Mardesic S. Fibrosis-Associated Signaling Molecules Are Differentially Expressed in Palmar Connective Tissues of Patients with Carpal Tunnel Syndrome and Dupuytren's Disease. Biomedicines. 2022;10(12). Epub 20221211. doi: 10.3390/biomedicines10123214. PubMed PMID: 36551969; PubMed Central PMCID: PMC9775445.

50. Docheva D, Vaerst B, Deiler S, Giunta RE, Schieker M, Volkmer E. [Alterations of cell phenotype in Dupuytren's disease--an in vitro analysis]. Handchir Mikrochir Plast Chir. 2012;44(2):59–66. Epub 2012/04/13. doi: 10.1055/s-0032-1309025. PubMed PMID: 22495955.

51. Devine MJ, Birsa N, Kittler JT. Miro sculpts mitochondrial dynamics in neuronal health and disease. Neurobiol Dis. 2016;90:27–34. Epub 20151219. doi: 10.1016/j.nbd.2015.12.008. PubMed PMID: 26707701.

52. Garcia-Martinez I, Garcia-Mesa Y, Garcia-Piqueras J, Martinez-Pubil A, Cobo JL, Feito J, et al. Sensory innervation of the human palmar aponeurosis in healthy individuals and patients with palmar fibromatosis. J Anat. 2021. Epub 20211208. doi: 10.1111/joa.13609. PubMed PMID: 34881452.

53. Nanchahal J, Chan JK. Treatments for early-stage Dupuytren's disease: an evidence-based approach. J Hand Surg Eur Vol. 2023:17531934221131373. Epub 20230113. doi: 10.1177/17531934221131373. PubMed PMID: 36638105.

54. Forrester HB, Temple-Smith P, Ham S, de Kretser D, Southwick G, Sprung CN. Genome-wide analysis using exon arrays demonstrates an important role for expression of extra-cellular matrix, fibrotic control and tissue remodelling genes in Dupuytren's disease. PLoS One. 2013;8(3):e59056. Epub 2013/04/05. doi: 10.1371/journal.pone.0059056. PubMed PMID: 23554969; PubMed Central PMCID: PMC3595223.

55. Augoff K, Tabola R, Kula J, Gosk J, Rutowski R. Epidermal growth factor receptor (EGF-R) in Dupuytren's disease. J Hand Surg Br. 2005;30(6):570–3. Epub 2005/08/02. doi: 10.1016/j.jhsb.2005.06.008. PubMed PMID: 16055243.

56. Joshi H, Morley SC. Cells under stress: The mechanical environment shapes inflammasome responses to danger signals. J Leukoc Biol. 2019;106(1):119–25. Epub 20190115. doi: 10.1002/JLB.3MIR1118-417R. PubMed PMID: 30645000; PubMed Central PMCID: PMC6784827.

57. Ali MK, Kim RY, Brown AC, Donovan C, Vanka KS, Mayall JR, et al. Critical role for iron accumulation in the pathogenesis of fibrotic lung disease. J Pathol. 2020;251(1):49–62. Epub 20200330. doi: 10.1002/path.5401. PubMed PMID: 32083318.

58. Ushijima M, Tsuneyoshi M, Enjoji M. Dupuytren type fibromatoses. A clinicopathologic study of 62 cases. Acta Pathol Jpn. 1984;34(5):991–1001. Epub 1984/09/01. doi: 10.1111/j.1440-1827.1984.tb07630.x. PubMed PMID: 6507097.

59. Dolmans GH, Werker PM, Hennies HC, Furniss D, Festen EA, Franke L, et al. Wnt signaling and Dupuytren's disease. N Engl J Med. 2011;365(4):307–17. Epub 2011/07/08. doi: 10.1056/NEJMoa1101029. PubMed PMID: 21732829.
