# Supplementary material for: Identification of novel plasma proteomic biomarkers of Dupuytren disease
Source: PLoS One. 2026 Mar 18;21(3):e0343733. doi: 10.1371/journal.pone.0343733 (PMC12998848; doi:10.1371/journal.pone.0343733)
Supplement: S2 Table — ID: Enriched category name. STR: Strength of protein-protein interaction (PPI). FDR: False Discovery Rate (Adjusted p-value). Overall PPI enrichment p-value: 0.000455. Figure 4 summarizes enriched pathways for individual genes. (DOCX) [file pone.0343733.s007.docx]

| **Category** | **ID** | **Description** | **STR** | **FDR** | **Genes of matching proteins in this network** |
| --- | --- | --- | --- | --- | --- |
| GO Function | GO:0005102 | Signaling receptor binding | 0.59 | 0.0084 | *C5, EDIL3, GUSB, PCSK9, CASP3, WFIKKN2, HSP90AA1, CRKL, CGB3, SYK, NRG4, TF, CALCB, CGB7, CGA, KNG1* |
| GO Function | GO:0004857 | Enzyme inhibitor activity | 0.87 | 0.0322 | *C5, PRKAR2B, CASP3, WFIKKN2, SOCS3, SERPINC1, YWHAB, KNG1* |
| GO Function | GO:0045309 | Protein phosphorylated amino acid binding | 1.41 | 0.0381 | *SOCS3, CRKL, YWHAB, SYK* |
| GO Component | GO:0005615 | Extracellular space | 0.5 | 5.52E-06 | *C5, DSG2, SCPEP1, C6, PRKAR2B, EDIL3, GUSB, PCSK9, PRSS1, WFIKKN2, SHMT1, HGS, HSP90AA1, CGB3, SERPINC1, KIAA1324, AKR1A1, YWHAB, POSTN, NRG4, TF, CPB1, CALCB, FKBP5, CGB7, OSCAR, CGA, KNG1* |
| GO Component | GO:0005576 | Extracellular region | 0.43 | 7.90E-06 | *C5, DSG2, SCPEP1, C6, PRKAR2B, EDIL3, GUSB, PCSK9, PRSS1, WFIKKN2, SHMT1, HGS, HSP90AA1, CGB3, SERPINC1, KIAA1324, AKR1A1, YWHAB, SCGN, POSTN, NRG4, TF, ACAN, CPB1, CALCB, FKBP5, CGB7, OSCAR, CGA, KNG1, BIN2* |
| GO Component | GO:0031982 | Vesicle | 0.38 | 0.0025 | *C5, DSG2, SCPEP1, C6, PRKAR2B, EDIL3, GUSB, PCSK9, RAB24, AOC3, SHMT1, HGS, HSP90AA1, SERPINC1, KIAA1324, AKR1A1, YWHAB, SYK, SCGN, USP8, TF, CPB1, FKBP5, OSCAR, KNG1, BIN2* |
| GO Component | GO:0070062 | Extracellular exosome | 0.5 | 0.0035 | *C5, DSG2, SCPEP1, C6, PRKAR2B, EDIL3, GUSB, SHMT1, HGS, HSP90AA1, SERPINC1, KIAA1324, AKR1A1, YWHAB, TF, FKBP5, OSCAR, KNG1* |
| GO Component | GO:0019897 | Extrinsic component of plasma membrane | 1.02 | 0.0327 | *PCSK9, CRKL, SYK, USP8, TF* |
| GO Component | GO:0062023 | Collagen-containing extracellular matrix | 0.8 | 0.0327 | *EDIL3, PRSS1, HSP90AA1, SERPINC1, POSTN, ACAN, KNG1* |
| KEGG | hsa04610 | Complement and coagulation cascades | 1.25 | 0.0301 | *C5, C6, SERPINC1, KNG1* |
| Reactome | HSA-162582 | Signal Transduction | 0.46 | 0.0148 | *C5, CSNK1G2, DSG2, PRKAR2B, SMAD1, MAP3K11, CASP3, SOCS3, HGS, HSP90AA1, CRKL, RHOT1, YWHAB, SYK, NRG4, USP8, CALCB, FKBP5, CGA, KNG1* |

**S2 Table. Functionally enriched pathways derived from the 54 differentially expressed proteins in the hypothesis-free analysis**. ID: Enriched category name. STR: Strength of protein-protein interaction (PPI). FDR: False Discovery Rate (Adjusted p-value). Overall PPI enrichment p-value: 0.000455. Figure 4 summarizes enriched pathways for individual genes.
