# Supplementary material for: Identification of novel plasma proteomic biomarkers of Dupuytren disease
Source: PLoS One. 2026 Mar 18;21(3):e0343733. doi: 10.1371/journal.pone.0343733 (PMC12998848; doi:10.1371/journal.pone.0343733)
Supplement: S3 Table — We combined three literature-based search strategies to identify candidate Dupuytren-related genes for hypothesis-based analytics. We first collected 2547 full-text publications from 1980 to 2023 that included at least three instances of the search terms “Dupuytren*” and/or “palmar fibroma*”. In the first strategy, we searched each publication for 4527 potential Dupuytren-related genes and their 33907 gene name aliases. This search identified 326 unique proteins or parent genes mentioned at least three times in at least one publication. The second method identified 117 selected collagen metabolism-related proteins or their parent genes that appeared at least once in at least one of these publications. The third method identified potentially DD-related single-nucleotide polymorphisms (SNPs) reported in these publications and compiled a list of 369 genes adjacent to these SNPs. We merged these three lists, resulting in 546 unique protein-coding genes, 328 of which matched proteins on the SomaScan panel, which contains 6995 protein-binding sites. Proteins expressed by these 328 genes were the targets of our hypothesis-based analysis. (DOCX) [file pone.0343733.s008.docx]

| *A1BG, A2M, ACAN, ACAT1, ACVR1B, ADAM12, ADAM15, ADAMTS3, ADH1B, ADK, AGA, AGT, AKR1C2, ALDH2, AMT, ANGPTL4, ANGPTL7, AOC3, APOB, ARHGEF10, ARHGEF2, ATXN3, AXIN2, AZGP1, B2M, BCL2, BDNF, BGN, BMP1, BMP6, BSG, C3, CADM1, CANT1, CARD18, CASP3, CASP8, CAT, CCL2, CCL5, CCN2, CCN4, CD34, CD4, CD40LG, CD44, CD68, CD8A, CDH1, CDH11, CDH13, CDH4, CDKN1A, CERT1, CFDP1, CFP, CHI3L1, CHRD, CHST6, CLU, CNTN2, CNTN6, COL10A1, COL11A2, COL13A1, COL15A1, COL18A1, COL1A1, COL20A1, COL23A1, COL25A1, COL28A1, COL2A1, COL3A1, COL5A1, COL6A1, COL6A2, COL6A3, COL6A5, COL8A1, COL9A1, COL9A3, COLGALT1, COLGALT2, COQ7, CSF2, CSMD1, CSNK1G2, CTHRC1, CTNNB1, CTSK, CXCL1, CXCL14, CXCL8, DAB2, DCN, DDR1, DDR2, DES, DKK1, DPP10, DPP4, DSCAML1, EED, EGF, EGFR, EGLN1, EGLN2, EGLN3, ELANE, ENG, EPDR1, EYS, F13A1, FAM20A, FBP1, FGF2, FGFR2, FKBP4, FMOD, FN1, FRZB, FST, G0S2, GCKR, GDF5, GH1, GJA1, GLB1, GPC1, GPR142, GPT, GPX3, GRIA4, HBEGF, HGF, HIF1A, HLA-C, HLA-G, HMGA2, HPRT1, HPX, HSD17B7, HSF1, HSPG2, ICAM1, IFNG, IGF1, IGF1R, IGF2, IGFBP6, IGFBP7, IKBKB, IL13, IL17A, IL1A, IL1B, IL6, INHBA, INS, INSR, IST1, ITGA11, ITGA2, ITGA4, ITGA5, ITGA6, ITGB1, KDR, KIN, KITLG, KNG1, KPNA2, KRT1, KRT34, LAMA3, LAMB1, LAMC2, LCN2, LOXL2, LOXL3, LRP5, LUM, LY75, MAP2K1, MAP4K5, MAPK3, MB, MECP2, MFAP5, MIF, MME, MMP1, MMP10, MMP12, MMP13, MMP14, MMP2, MMP20, MMP3, MMP7, MMP8, MMP9, MOCOS, MYC, NCALD, NCL, NDE1, NECAB1, NFKB1, NGF, NGFR, NID1, NME8, NOG, NRG1, NRXN1, NT5E, NTM, OSTF1, P3H1, P4HA1, P4HA2, P4HB, PCNA, PCOLCE, PCOLCE2, PCSK1N, PCSK7, PDGFA, PDLIM5, PECAM1, PIK3AP1, PLAT, PLCG1, PLG, PLOD2, PLOD3, PLXDC2, POLD4, POSTN, PPARA, PPIB, PRKCA, PROS1, PSTPIP1, PTGDS, PTGS2, PTK2, PTN, PTPRD, RAB31, RBFOX1, RND3, ROR2, RSPO2, S100A6, SERPINE1, SERPINH1, SFRP1, SFRP4, SH2D1A, SH3BP2, SH3GL2, SIRPB1, SLC14A2, SLMAP, SMAD1, SMAD2, SMAD3, SMAD4, SOD1, SOD2, SORCS3, SPARC, SPOCK3, SPP1, STAT1, STAT3, STATH, STIP1, STUB1, SUMO4, TAC1, TAGLN, TBK1, TCF4, TF, TFPI2, TGFB1, TGFB2, TGFB3, TGFBR1, TGFBR2, THBS2, THBS4, THY1, TIMP1, TIMP2, TIMP3, TMEM132A, TNC, TNF, TNFRSF1B, TNIP1, TNXB, TP53, TPI1, TXN, UBE2D3, UBE2K, USP8, UST, VAPA, VCAM1, VDR, VEGFA, VIM, VTN, WIF1, WNK1, WNT11, WNT3A, WNT5A, WWOX, YAP1, YWHAZ, ZNF264* |
| --- |

**S3 Table. 328 protein-coding genes in the SomaLogic hypothesis-based analysis**. We combined three literature-based search strategies to identify candidate Dupuytren-related genes for hypothesis-based analytics. We first collected 2547 full-text publications from 1980 to 2023 that included at least three instances of the search terms "Dupuytren*" and/or "palmar fibroma*". In the first strategy, we searched each publication for 4527 potential Dupuytren-related genes and their 33907 gene name aliases. This search identified 326 unique proteins or parent genes mentioned at least three times in at least one publication. The second method identified 117 selected collagen metabolism-related proteins or their parent genes that appeared at least once in at least one of these publications. The third method identified potentially DD-related single-nucleotide polymorphisms (SNPs) reported in these publications and compiled a list of 369 genes adjacent to these SNPs. We merged these three lists, resulting in 546 unique protein-coding genes, 328 of which matched proteins on the SomaScan panel, which contains 6995 protein-binding sites. Proteins expressed by these 328 genes were the targets of our hypothesis-based analysis.
