# Supplementary material for: Identification of novel plasma proteomic biomarkers of Dupuytren disease
Source: PLoS One. 2026 Mar 18;21(3):e0343733. doi: 10.1371/journal.pone.0343733 (PMC12998848; doi:10.1371/journal.pone.0343733)
Supplement: S4 Table — Of the 328 aptamers in the Hypothesis-based analysis, 23 had statistically significant differences between DD and control, with nine overexpressed and fourteen underexpressed after adjusting for multiple comparisons. Nine of these genes appeared in hypothesis-free and hypothesis-based analyses, as indicated by *. Plain italics indicate an indirect DD relationship, if any, to the protein; bold italics indicate a direct relationship between published DD findings and the protein. In the Notes column, “Referred to” means the referenced publication referred to the gene by an alias name. Exp: DD expression compared to controls. FDR: False Discovery Rate (Adjusted p-value) of expression difference. Nodes: number of pathway analysis connections (nodes) of this protein to others in this group with a medium confidence interaction score (0.400) and an FDR<=0.05. Notes: potential relationship to DD biology. Cat: categories of possible relationships to DD: 1. Apoptosis and senescence (2 genes); 2. Bioinformatics (7 genes); 3. Clinical and demographic (3 genes); 4. Extracellular matrix (9 genes); 5. Fibroblast and myofibroblast cytoskeleton, membrane, and motility (6 genes); 6. Fibroblast and myofibroblast differentiation and transcriptome (5 genes); 7. Vascular and perivascular (4 genes). (DOCX) [file pone.0343733.s009.docx]

| **Gene** | **Express** | **p-val** | **FDR** | **Nodes** | **Notes** | **Cat** |
| --- | --- | --- | --- | --- | --- | --- |
| *ACAN** | Up | 0.001148 | 0.074 | 3 | Aggrecan core protein 2; Proteoglycan, binds to hyaluronic acid. ***ACAN is near DD-related SNP rs6496519 and is dysregulated in DD transcriptomic profiling***. Extracellular matrix interactions are key components of DD biology. [1, 2, 3] | 2, 4 |
| *AOC3** | Up | 0.000845 | 0.074 | 0 | Membrane primary amine oxidase; cell adhesion protein. Increased ***AOC3 levels in small vessel endothelium cells in DD-affected tissues***. (referred to by alias VAP-1), *DD is associated with local microvascular inflammation, thrombosis, and endothelial leucocyte adhesion.* [4, 5] | 7 |
| *CASP3** | Down | 0.001603 | 0.075 | 8 | Caspase-3 subunit p12; Involved in apoptosis. ***PPI Network analysis projects CASP3 involvement in DD****. Apoptosis is dysregulated in DD.* ***CASP3 is dysregulated in DD transcriptomic profiling***. [1, 6, 7] | 1, 2 |
| *COL1A1* | Up | 0.010705 | 0.211 | 7 | Collagen alpha-1(I) chain. *Homotrimer Collagen (a1) is increased in DD and resistant to enzymatic degradation. Differential expression in DD cell culture.* [1, 8, 9] | 4 |
| *CSMD1* | Up | 0.007311 | 0.191 | 0 | CUB and sushi domain-containing protein 1; Potential suppressor of squamous cell carcinomas; *CSMD1* variant associated with reduced severity of post-burn hypertrophic scarring. *Hypertrophic scar shares some ECM, microvascular, and mechanical load effects with DD*. [10-13] | 5, 7 |
| *CSNK1G2** | Down | 0.001222 | 0.074 | 0 | Casein kinase I isoform gamma-2; Serine/threonine-protein kinase. Participates in WNT signaling. WNT expression is dysregulated in DD; dysregulated in DD transcriptomic profiling. ***Pathway analysis projects differential CSNK1G2 expression in both visibly affected and normal-appearing tissues in DD vs. control***. [1, 14, 15] | 2 |
| *DAB2* | Down | 0.009245 | 0.211 | 1 | Disabled homolog 2; regulates cell membrane adhesion complex disassembly during cell migration. *Fibroblast adhesion and migration processes are abnormal in DD*. [16, 17] | 5 |
| *DDR2* | Up | 0.010921 | 0.211 | 3 | Discoidin domain-containing receptor 2; Tyrosine kinase that functions as a cell surface receptor for fibrillar collagen and regulates cell differentiation, remodeling of the extracellular matrix, cell migration, and cell proliferation. Regulates extracellular matrix remodeling by up-regulating the collagenases MMP1, MMP2, and MMP13. Promotes fibroblast migration and proliferation. *All of these processes are abnormal in DD.* ***DDR2 is near the DD-related SNP rs17433710***. [2, 3, 16] | 2, 4, 5 |
| *HPX* | Up | 0.013002 | 0.229 | 3 | Hemopexin; required for MMP1 activity. *MMP1 plays a key role in DD biology*. [18, 19] | 4 |
| *KNG1** | Up | 0.000490 | 0.069 | 3 | Kininogen1; Alternative splicing produces high molecular weight kininogen (HMWK) and low molecular weight kininogen (LMWK). HMWK inhibits thrombin- and plasmin-induced thrombocyte aggregation; stimulates the release of other mediators of inflammation; causes vasodilation and increases vascular permeability; the bradykinin B2 receptor contributes to endothelial inflammation. *DD is associated with local microvascular inflammation, thrombosis, and endothelial leucocyte adhesion*. [4, 20] | 7 |
| *LCN2* | Down | 0.001910 | 0.081 | 3 | LCN2 is an iron-ion binding protein; involved in multiple processes including innate immunity and ferroptosis. *Hemosiderin deposition occurs in the early cellular stage of DD*. LCN2 protects MMP9 from degradation. *MMP9 activates TGFB*. (referred to by alias LGA2) [21-24] | 3, 4 |
| *MAP2K1* | Down | 0.003473 | 0.133 | 4 | Dual specificity mitogen-activated protein kinase kinase 1; MAP2K1 phosphorylation from mechanical stretch increases TGFB expression. Mechanical stretch of myofibroblasts attached to a stiff extracellular matrix activates latent TGFB1. *Increasing ECM stiffness increases smooth muscle actin expression and reduces migration of DD fibroblasts compared to control fibroblasts*. [16, 25, 26] | 4, 5 |
| *PLAT* | Down | 0.006181 | 0.186 | 2 | Tissue-type plasminogen activator chain A; converts plasminogen to plasmin, which, in turn, activates TGFB. *TGFB1 and TGFB2 are involved in key DD-related pathways*. [27] | 6 |
| *POSTN** | Up | 0.000312 | 0.066 | 6 | Periostin; Induces cell attachment and spreading and plays a role in cell adhesion. Enhances incorporation of BMP1 in the fibronectin matrix of connective tissues, and subsequent proteolytic activation of lysyl oxidase LOX. *Increased LOX activity in Dupuytren tissue.* ***Increased POSTN expression in DD fibroblasts and in sweat glands of DD-adjacent skin***. [1, 28-31] | 4, 6 |
| *PRKCA* | Down | 0.009837 | 0.211 | 4 | Protein kinase C alpha type; involved in the regulation of cell proliferation, apoptosis, differentiation, migration, and adhesion; multiple protein-protein interactions in DD network biology. (referred to by alias *PRKACA*). [32] | 1, 5 |
| *SERPINH1* | Down | 0.011476 | 0.211 | 3 | SERPINH1; collagen-binding protein that may affect the expression of collagen III. ***Increased SERPINH1 expression in DD tissue***. (referred to by alias *HSP47*). [33, 34] | 4, 5 |
| *SFRP4* | Down | 0.004311 | 0.152 | 2 | Secreted frizzled-related protein 4; inhibits WNT/frizzled receptor signaling pathway. ***Near DD-related SNPs rs16879765 and rs17171229.*** [35] | 2 |
| *SMAD1** | Down | 0.001275 | 0.074 | 1 | Mothers against decapentaplegic homolog 1; Transcriptional modulator activated by BMP (bone morphogenetic proteins) type 1 receptor kinase. SMAD1/5/9 pathway action is antifibrotic in multiple organ fibroses. ***SMAD1 protein expression is normal in DD tissues, but intracellular SMAD1 mRNA expression is reduced in DD fibroblasts.*** [36] | 2, 6 |
| *STAT1* | Down | 0.006707 | 0.189 | 4 | Signal transducer and activator of transcription 1-alpha/beta; mediates cellular responses to interferons and other cytokines and other growth factors; ***significantly reduced STAT1 expression in DD cell cultures.*** [37] | 6 |
| *STAT3* | Down | 0.005622 | 0.182 | 9 | Signal transducer and activator of transcription 3; Signal transducer and transcription activator that mediates cellular responses to interleukins and growth factors. ***STAT3 is near Dupuytren-associated risk alleles rs16879765 and rs17171229 risk alleles and is associated with phosphorylated STAT3 in cultured DD fibroblasts***. [35] | 6 |
| *TF** | Up | 0.000025 | 0.010 | 3 | Serotransferrin; Responsible for the transport of iron from sites of absorption and heme degradation; iron levels are increased in the lung tissues of patients with idiopathic pulmonary fibrosis; exogenous iron increases human lung fibroblast proliferation and cytokine responses. *Local tissue iron deposition occurs in the early cellular stage of DD*. [22, 38] | 4, 7 |
| *USP8** | Down | 0.001398 | 0.074 | 1 | Ubiquitin carboxyl-terminal hydrolase 8; USP8 pathway is essential for WNT/β-catenin signaling. WNT signaling pathways are central in DD biology. ***USP8 is under-expressed in DD Fibroblasts by Genome-wide exon expression profiles***. USP8 may exert protective influences against aging. *DD prevalence is age-related*. [37, 39] | 2, 3 |
| *YWHAZ* | Down | 0.007679 | 0.191 | 4 | 14-3-3 protein zeta/delta; Increased expression in hypertrophic scars; hypertrophic scar shares some ECM, microvascular, and mechanical load effects with DD. *Pathway analysis associates YWHAZ with BRAF. BRAF inhibitors provoke DD-like clinical changes.* [12, 40-42] | 3 |

**S4 Table. 23 Differentially expressed genes in the Hypothesis-based analysis**. Of the 328 aptamers in the Hypothesis-based analysis, 23 had statistically significant differences between DD and control, with nine overexpressed and fourteen underexpressed after adjusting for multiple comparisons. Nine of these genes appeared in hypothesis-free and hypothesis-based analyses, as indicated by *. *Plain italics* indicate an indirect DD relationship, if any, to the protein; ***bold italics*** indicate a direct relationship between published DD findings and the protein. In the Notes column, "Referred to" means the referenced publication referred to the gene by an alias name. Exp: DD expression compared to controls. FDR: False Discovery Rate (Adjusted p-value) of expression difference. Nodes: number of pathway analysis connections (nodes) of this protein to others in this group with a medium confidence interaction score (0.400) and an FDR<=0.05. Notes: potential relationship to DD biology. Cat: categories of possible relationships to DD: 1. Apoptosis and senescence (2 genes); 2. Bioinformatics (7 genes); 3. Clinical and demographic (3 genes); 4. Extracellular matrix (9 genes); 5. Fibroblast and myofibroblast cytoskeleton, membrane, and motility (6 genes); 6. Fibroblast and myofibroblast differentiation and transcriptome (5 genes); 7. Vascular and perivascular (4 genes).

**S4 Table References**

1. Rehman S, Salway F, Stanley JK, Ollier WE, Day P, Bayat A. Molecular phenotypic descriptors of Dupuytren's disease defined using informatics analysis of the transcriptome. J Hand Surg Am. 2008;33(3):359–72. Epub 2008/03/18. doi: 10.1016/j.jhsa.2007.11.010. PubMed PMID: 18343292.

2. Ng M, Thakkar D, Southam L, Werker P, Ophoff R, Becker K, et al. A Genome-wide Association Study of Dupuytren Disease Reveals 17 Additional Variants Implicated in Fibrosis. Am J Hum Genet. 2017;101(3):417–27. Epub 2017/09/09. doi: 10.1016/j.ajhg.2017.08.006. PubMed PMID: 28886342; PubMed Central PMCID: PMC5591021.

3. O’Gorman DB. The Extracellular Matrix in Dupuytren Disease. Dupuytren Disease and Related Diseases - The Cutting Edge 2017. p. 43–54.

4. Mayerl C, Del Frari B, Parson W, Boeck G, Piza-Katzer H, Wick G, et al. Characterisation of the inflammatory response in Dupuytren's disease. J Plast Surg Hand Surg. 2016;50(3):171–9. Epub 2016/02/09. doi: 10.3109/2000656X.2016.1140054. PubMed PMID: 26852784.

5. Pannecoeck R, Serruys D, Benmeridja L, Delanghe JR, van Geel N, Speeckaert R, et al. Vascular adhesion protein-1: Role in human pathology and application as a biomarker. Crit Rev Clin Lab Sci. 2015;52(6):284–300. Epub 20150818. doi: 10.3109/10408363.2015.1050714. PubMed PMID: 26287391.

6. Stocks M, Walter AS, Akova E, Gauglitz G, Aszodi A, Boecker W, et al. RNA-seq unravels distinct expression profiles of keloids and Dupuytren's disease. Heliyon. 2024;10(1):e23681. Epub 20231213. doi: 10.1016/j.heliyon.2023.e23681. PubMed PMID: 38187218; PubMed Central PMCID: PMC10770622.

7. Wilutzky B, Berndt A, Katenkamp D, Koshmehl H. Programmed cell death in nodular palmar fibromatosis (Morbus Dupuytren). Histol Histopathol. 1998;13(1):67–72. Epub 1998/02/26. doi: 10.14670/HH-13.67. PubMed PMID: 9476635.

8. Williamson K, Cooper G, Lee KJ, Beamish EL, Simpson D, Carter A, et al. Active synthesis of type I collagen homotrimer in Dupuytren’s fibrosis is unaffected by anti-TNF-α treatment. bioRxiv preprint 2023. doi: 10.1101/2020.07.13.195107.

9. Han S, Makareeva E, Kuznetsova NV, DeRidder AM, Sutter MB, Losert W, et al. Molecular mechanism of type I collagen homotrimer resistance to mammalian collagenases. J Biol Chem. 2010;285(29):22276–81. Epub 2010/05/14. doi: 10.1074/jbc.M110.102079. PubMed PMID: 20463013; PubMed Central PMCID: PMC2903388.

10. Verjee LS, Midwood K, Davidson D, Essex D, Sandison A, Nanchahal J. Myofibroblast distribution in Dupuytren's cords: correlation with digital contracture. J Hand Surg Am. 2009;34(10):1785–94. Epub 2009/11/17. doi: 10.1016/j.jhsa.2009.08.005. PubMed PMID: 19910144.

11. Kischer CW, Speer DP. Microvascular changes in Dupuytren’s contracture. J Hand Surg Am. 1984;9A(1):58–62. doi: 10.1016/s0363-5023(84)80185-9. PubMed PMID: 6693745.

12. Aarabi S, Bhatt KA, Shi Y, Paterno J, Chang EI, Loh SA, et al. Mechanical load initiates hypertrophic scar formation through decreased cellular apoptosis. FASEB J. 2007;21(12):3250–61. Epub 2007/05/17. doi: 10.1096/fj.07-8218com. PubMed PMID: 17504973.

13. Sood RF, Hocking AM, Muffley LA, Ga M, Honari S, Reiner AP, et al. Genome-wide Association Study of Postburn Scarring Identifies a Novel Protective Variant. Ann Surg. 2015;262(4):563–9. doi: 10.1097/SLA.0000000000001439. PubMed PMID: 26366535; PubMed Central PMCID: PMC9632222.

14. van Beuge MM, Ten Dam EJ, Werker PM, Bank RA. Wnt pathway in Dupuytren disease: connecting profibrotic signals. Transl Res. 2015;166(6):762–71 e3. Epub 2015/10/17. doi: 10.1016/j.trsl.2015.09.006. PubMed PMID: 26470681.

15. Satish L, LaFramboise WA, Johnson S, Vi L, Njarlangattil A, Raykha C, et al. Fibroblasts from phenotypically normal palmar fascia exhibit molecular profiles highly similar to fibroblasts from active disease in Dupuytren's Contracture. BMC Med Genomics. 2012;5:15. Epub 2012/05/09. doi: 10.1186/1755-8794-5-15. PubMed PMID: 22559715; PubMed Central PMCID: PMC3375203.

16. Viji Babu PK, Rianna C, Belge G, Mirastschijski U, Radmacher M. Mechanical and migratory properties of normal, scar, and Dupuytren's fibroblasts. J Mol Recognit. 2018;31(9):e2719. Epub 2018/04/28. doi: 10.1002/jmr.2719. PubMed PMID: 29701269.

17. Teckchandani A, Toida N, Goodchild J, Henderson C, Watts J, Wollscheid B, et al. Quantitative proteomics identifies a Dab2/integrin module regulating cell migration. J Cell Biol. 2009;186(1):99–111. Epub 20090706. doi: 10.1083/jcb.200812160. PubMed PMID: 19581412; PubMed Central PMCID: PMC2712992.

18. Itoh Y, Ng M, Wiberg A, Inoue K, Hirata N, Paiva KBS, et al. A common SNP risk variant MT1-MMP causative for Dupuytren's disease has a specific defect in collagenolytic activity. Matrix Biol. 2021;97:20–39. Epub 2021/02/17. doi: 10.1016/j.matbio.2021.02.003. PubMed PMID: 33592276.

19. Johnston P, Chojnowski AJ, Davidson RK, Riley GP, Donell ST, Clark IM. A complete expression profile of matrix-degrading metalloproteinases in Dupuytren's disease. J Hand Surg Am. 2007;32(3):343–51. Epub 2007/03/06. doi: 10.1016/j.jhsa.2006.12.010. PubMed PMID: 17336841.

20. Terzuoli E, Corti F, Nannelli G, Giachetti A, Donnini S, Ziche M. Bradykinin B2 Receptor Contributes to Inflammatory Responses in Human Endothelial Cells by the Transactivation of the Fibroblast Growth Factor Receptor FGFR-1. Int J Mol Sci. 2018;19(9). Epub 20180906. doi: 10.3390/ijms19092638. PubMed PMID: 30200598; PubMed Central PMCID: PMC6163484.

21. Romejko K, Markowska M, Niemczyk S. The Review of Current Knowledge on Neutrophil Gelatinase-Associated Lipocalin (NGAL). Int J Mol Sci. 2023;24(13). Epub 20230621. doi: 10.3390/ijms241310470. PubMed PMID: 37445650; PubMed Central PMCID: PMC10341718.

22. Ushijima M, Tsuneyoshi M, Enjoji M. Dupuytren type fibromatoses. A clinicopathologic study of 62 cases. Acta Pathol Jpn. 1984;34(5):991–1001. Epub 1984/09/01. doi: 10.1111/j.1440-1827.1984.tb07630.x. PubMed PMID: 6507097.

23. D'Amico F, Candido S, Libra M. Interaction between matrix metalloproteinase-9 (MMP-9) and neutrophil gelatinase-associated lipocalin (NGAL): A recent evolutionary event in primates. Dev Comp Immunol. 2021;116:103933. Epub 20201124. doi: 10.1016/j.dci.2020.103933. PubMed PMID: 33245981.

24. Luo L, Deng L, Chen Y, Ding R, Li X. Identification of Lipocalin 2 as a Ferroptosis-Related Key Gene Associated with Hypoxic-Ischemic Brain Damage via STAT3/NF-kappaB Signaling Pathway. Antioxidants (Basel). 2023;12(1). Epub 20230112. doi: 10.3390/antiox12010186. PubMed PMID: 36671050; PubMed Central PMCID: PMC9854551.

25. Wipff PJ, Hinz B. Integrins and the activation of latent transforming growth factor beta1 - an intimate relationship. Eur J Cell Biol. 2008;87(8-9):601–15. Epub 2008/03/18. doi: 10.1016/j.ejcb.2008.01.012. PubMed PMID: 18342983.

26. Dong G, Huang X, Jiang S, Ni L, Chen S. Simvastatin Mitigates Apoptosis and Transforming Growth Factor-Beta Upregulation in Stretch-Induced Endothelial Cells. Oxid Med Cell Longev. 2019;2019:6026051. Epub 20191217. doi: 10.1155/2019/6026051. PubMed PMID: 31934265; PubMed Central PMCID: PMC6942893.

27. Lyons RM, Gentry LE, Purchio AF, Moses HL. Mechanism of activation of latent recombinant transforming growth factor beta 1 by plasmin. J Cell Biol. 1990;110(4):1361–7. doi: 10.1083/jcb.110.4.1361. PubMed PMID: 2139036; PubMed Central PMCID: PMC2116088.

28. Verhoekx JSN, Verjee LS, Izadi D, Chan JKK, Nicolaidou V, Davidson D, et al. Isometric contraction of Dupuytren's myofibroblasts is inhibited by blocking intercellular junctions. J Invest Dermatol. 2013;133(12):2664–71. Epub 2013/05/09. doi: 10.1038/jid.2013.219. PubMed PMID: 23652794.

29. Hamamoto H, Ueba Y, Sudo Y, Sanada H, Yamamuro T, Takeda T. Dupuytren's contracture: morphological and biochemical changes in palmar aponeurosis. Hand. 1982;14(3):237–47. Epub 1982/10/01. doi: 10.1016/s0072-968x(82)80056-9. PubMed PMID: 6130030.

30. Shih B, Brown JJ, Armstrong DJ, Lindau T, Bayat A. Differential gene expression analysis of subcutaneous fat, fascia, and skin overlying a Dupuytren's disease nodule in comparison to control tissue. Hand (N Y). 2009;4(3):294–301. Epub 2009/02/03. doi: 10.1007/s11552-009-9164-0. PubMed PMID: 19184239; PubMed Central PMCID: PMC2724615.

31. Tripkovic I, Ogorevc M, Vukovic D, Saraga-Babic M, Mardesic S. Fibrosis-Associated Signaling Molecules Are Differentially Expressed in Palmar Connective Tissues of Patients with Carpal Tunnel Syndrome and Dupuytren's Disease. Biomedicines. 2022;10(12). Epub 20221211. doi: 10.3390/biomedicines10123214. PubMed PMID: 36551969; PubMed Central PMCID: PMC9775445.

32. Flevaris P, Vaughan D. The Role of Plasminogen Activator Inhibitor Type-1 in Fibrosis. Semin Thromb Hemost. 2017;43(2):169–77. Epub 20160824. doi: 10.1055/s-0036-1586228. PubMed PMID: 27556351.

33. Howard JC, Varallo VM, Ross DC, Faber KJ, Roth JH, Seney S, et al. Wound healing-associated proteins Hsp47 and fibronectin are elevated in Dupuytren's contracture. J Surg Res. 2004;117(2):232–8. Epub 2004/03/30. doi: 10.1016/j.jss.2004.01.013. PubMed PMID: 15047128.

34. Hosokawa N, Hohenadl C, Satoh M, Kuhn K, Nagata K. HSP47, a collagen-specific molecular chaperone, delays the secretion of type III procollagen transfected in human embryonic kidney cell line 293: a possible role for HSP47 in collagen modification. J Biochem. 1998;124(3):654–62. doi: 10.1093/oxfordjournals.jbchem.a022162. PubMed PMID: 9722680.

35. Kida H, Jiang JJ, Matsui Y, Takahashi I, Hasebe R, Kawamura D, et al. Dupuytren's contracture-associated SNPs increase SFRP4 expression in nonimmune cells including fibroblasts to enhance inflammation development. Int Immunol. 2023. Epub 20230131. doi: 10.1093/intimm/dxad004. PubMed PMID: 36719100.

36. Krause C, Kloen P, Ten Dijke P. Elevated transforming growth factor beta and mitogen-activated protein kinase pathways mediate fibrotic traits of Dupuytren's disease fibroblasts. Fibrogenesis Tissue Repair. 2011;4(1):14. Epub 2011/06/30. doi: 10.1186/1755-1536-4-14. PubMed PMID: 21711521; PubMed Central PMCID: PMC3148569.

37. Forrester HB, Temple-Smith P, Ham S, de Kretser D, Southwick G, Sprung CN. Genome-wide analysis using exon arrays demonstrates an important role for expression of extra-cellular matrix, fibrotic control and tissue remodelling genes in Dupuytren's disease. PLoS One. 2013;8(3):e59056. Epub 2013/04/05. doi: 10.1371/journal.pone.0059056. PubMed PMID: 23554969; PubMed Central PMCID: PMC3595223.

38. Ali MK, Kim RY, Brown AC, Donovan C, Vanka KS, Mayall JR, et al. Critical role for iron accumulation in the pathogenesis of fibrotic lung disease. J Pathol. 2020;251(1):49–62. Epub 20200330. doi: 10.1002/path.5401. PubMed PMID: 32083318.

39. Dolmans GH, Werker PM, Hennies HC, Furniss D, Festen EA, Franke L, et al. Wnt signaling and Dupuytren's disease. N Engl J Med. 2011;365(4):307–17. Epub 2011/07/08. doi: 10.1056/NEJMoa1101029. PubMed PMID: 21732829.

40. Vandersleyen V, Grosber M, Wilgenhof S, De Kock J, Neyns B, Gutermuth J. Vemurafenib-associated Dupuytren- and Ledderhose palmoplantar fibromatosis in metastatic melanoma patients. J Eur Acad Dermatol Venereol. 2016;30(7):1133–5. Epub 20150824. doi: 10.1111/jdv.13268. PubMed PMID: 26303964.

41. Li Z, Ivanov AA, Su R, Gonzalez-Pecchi V, Qi Q, Liu S, et al. The OncoPPi network of cancer-focused protein-protein interactions to inform biological insights and therapeutic strategies. Nat Commun. 2017;8:14356. Epub 20170216. doi: 10.1038/ncomms14356. PubMed PMID: 28205554; PubMed Central PMCID: PMC5316855.

42. van den Broek LJ, van der Veer WM, de Jong EH, Gibbs S, Niessen FB. Suppressed inflammatory gene expression during human hypertrophic scar compared to normotrophic scar formation. Exp Dermatol. 2015;24(8):623–9. Epub 20150526. doi: 10.1111/exd.12739. PubMed PMID: 25939875.
