# Supplementary material for: Identification of novel plasma proteomic biomarkers of Dupuytren disease
Source: PLoS One. 2026 Mar 18;21(3):e0343733. doi: 10.1371/journal.pone.0343733 (PMC12998848; doi:10.1371/journal.pone.0343733)
Supplement: S5 Table — ID: Enriched category name. STR: Strength of protein-protein interaction (PPI). FDR: False Discovery Rate (Adjusted p-value). Overall PPI enrichment p-value: 1.35E-07. Hypothesis-based candidate protein preselection may influence enrichment results. Figure 6 summarizes enriched pathways for individual genes. (DOCX) [file pone.0343733.s010.docx]

| **Category** | **ID** | **Description** | **STR** | **FDR** | **Genes of matching proteins in this network** |
| --- | --- | --- | --- | --- | --- |
| GO Process | GO:0050896 | Response to stimulus | 0.38 | 0.0004 | *PLAT, COL1A1, CSNK1G2, STAT3, HPX, MAP2K1, SMAD1, CASP3, AOC3, DAB2, STAT1, DDR2, LCN2, POSTN, YWHAZ, USP8, TF, PRKCA, SFRP4, SERPINH1, CSMD1, KNG1* |
| GO Process | GO:0042221 | Response to chemical | 0.56 | 0.0004 | *PLAT, COL1A1, STAT3, HPX, MAP2K1, SMAD1, CASP3, AOC3, DAB2, STAT1, DDR2, POSTN, USP8, TF, PRKCA, SFRP4, SERPINH1* |
| GO Process | GO:0009725 | Response to hormone | 1.01 | 0.0005 | *PLAT, COL1A1, STAT3, CASP3, DAB2, STAT1, DDR2, USP8, SFRP4* |
| GO Process | GO:0010033 | Response to organic substance | 0.65 | 0.0008 | *PLAT, COL1A1, STAT3, HPX, SMAD1, CASP3, DAB2, STAT1, DDR2, POSTN, USP8, PRKCA, SFRP4, SERPINH1* |
| GO Process | GO:0045597 | Positive regulation of cell differentiation | 0.94 | 0.0010 | *COL1A1, STAT3, MAP2K1, SMAD1, DAB2, STAT1, DDR2, PRKCA, SFRP4* |
| GO Process | GO:0007166 | Cell surface receptor signaling pathway | 0.7 | 0.0016 | *PLAT, COL1A1, CSNK1G2, STAT3, HPX, SMAD1, CASP3, DAB2, STAT1, DDR2, TF, SFRP4* |
| GO Process | GO:0009719 | Response to endogenous stimulus | 0.8 | 0.0016 | *PLAT, COL1A1, STAT3, SMAD1, CASP3, DAB2, STAT1, DDR2, USP8, SFRP4* |
| GO Process | GO:0009888 | Tissue development | 0.74 | 0.0016 | *COL1A1, MAP2K1, SMAD1, CASP3, DAB2, STAT1, DDR2, POSTN, ACAN, SERPINH1, CSMD1* |
| GO Process | GO:0030177 | Positive regulation of WNT signaling pathway | 1.48 | 0.0016 | *COL1A1, CSNK1G2, DAB2, USP8, SFRP4* |
| GO Process | GO:0030199 | Collagen fibril organization | 1.76 | 0.0016 | *COL1A1, DDR2, ACAN, SERPINH1* |
| GO Process | GO:0065008 | Regulation of biological quality | 0.55 | 0.0016 | *PLAT, STAT3, HPX, SMAD1, CASP3, DAB2, STAT1, LCN2, YWHAZ, USP8, TF, PRKCA, SFRP4, CSMD1, KNG1* |
| GO Process | GO:0071363 | Cellular response to growth factor stimulus | 1.1 | 0.0016 | *COL1A1, STAT3, SMAD1, CASP3, DAB2, DDR2, USP8* |
| GO Process | GO:0071560 | Cellular response to transforming growth factor beta stimulus | 1.45 | 0.0016 | *COL1A1, STAT3, SMAD1, DAB2, DDR2* |
| GO Process | GO:0007154 | Cell communication | 0.45 | 0.0022 | *PLAT, COL1A1, CSNK1G2, STAT3, HPX, MAP2K1, SMAD1, CASP3, DAB2, STAT1, DDR2, POSTN, YWHAZ, USP8, TF, PRKCA, SFRP4* |
| GO Process | GO:0051239 | Regulation of multicellular organismal process | 0.61 | 0.0022 | *PLAT, COL1A1, STAT3, MAP2K1, SMAD1, DAB2, STAT1, DDR2, LCN2, TF, PRKCA, SFRP4, KNG1* |
| GO Process | GO:0071495 | Cellular response to endogenous stimulus | 0.84 | 0.0022 | *PLAT, COL1A1, STAT3, SMAD1, CASP3, DAB2, STAT1, DDR2, USP8* |
| GO Process | GO:0006950 | Response to stress | 0.55 | 0.0025 | *PLAT, COL1A1, STAT3, HPX, MAP2K1, SMAD1, CASP3, AOC3, STAT1, DDR2, LCN2, TF, SERPINH1, KNG1* |
| GO Process | GO:0009967 | Positive regulation of signal transduction | 0.75 | 0.0025 | *COL1A1, CSNK1G2, STAT3, HPX, MAP2K1, DAB2, DDR2, USP8, PRKCA, SFRP4* |
| GO Process | GO:0010646 | Regulation of cell communication | 0.55 | 0.0025 | *PLAT, COL1A1, CSNK1G2, STAT3, HPX, MAP2K1, DAB2, STAT1, DDR2, POSTN, YWHAZ, USP8, PRKCA, SFRP4* |
| GO Process | GO:0014070 | Response to organic cyclic compound | 0.9 | 0.0025 | *PLAT, COL1A1, STAT3, SMAD1, CASP3, DAB2, STAT1, USP8* |
| GO Process | GO:0023051 | Regulation of signaling | 0.55 | 0.0025 | *PLAT, COL1A1, CSNK1G2, STAT3, HPX, MAP2K1, DAB2, STAT1, DDR2, POSTN, YWHAZ, USP8, PRKCA, SFRP4* |
| GO Process | GO:0048583 | Regulation of response to stimulus | 0.51 | 0.0025 | *PLAT, COL1A1, CSNK1G2, STAT3, HPX, MAP2K1, DAB2, STAT1, DDR2, POSTN, YWHAZ, USP8, PRKCA, SFRP4, KNG1* |
| GO Process | GO:0007165 | Signal transduction | 0.46 | 0.0030 | *PLAT, COL1A1, CSNK1G2, STAT3, HPX, MAP2K1, SMAD1, CASP3, DAB2, STAT1, DDR2, YWHAZ, USP8, TF, PRKCA, SFRP4* |
| GO Process | GO:0060348 | Bone development | 1.3 | 0.0030 | *COL1A1, SMAD1, DDR2, SFRP4, SERPINH1* |
| GO Process | GO:0009966 | Regulation of signal transduction | 0.57 | 0.0032 | *COL1A1, CSNK1G2, STAT3, HPX, MAP2K1, DAB2, STAT1, DDR2, POSTN, YWHAZ, USP8, PRKCA, SFRP4* |
| GO Process | GO:0007167 | Enzyme-linked receptor protein signaling pathway | 0.97 | 0.0035 | *PLAT, COL1A1, STAT3, SMAD1, CASP3, DDR2, TF* |
| GO Process | GO:0090263 | Positive regulation of canonical Wnt signaling pathway | 1.51 | 0.0039 | *COL1A1, CSNK1G2, USP8, SFRP4* |
| GO Process | GO:0016310 | Phosphorylation | 0.85 | 0.0045 | *CSNK1G2, STAT3, HPX, MAP2K1, SMAD1, DDR2, YWHAZ, PRKCA* |
| GO Process | GO:0048584 | Positive regulation of response to stimulus | 0.65 | 0.0045 | *PLAT, COL1A1, CSNK1G2, STAT3, HPX, MAP2K1, DAB2, DDR2, USP8, PRKCA, SFRP4* |
| GO Process | GO:0060828 | Regulation of canonical Wnt signaling pathway | 1.23 | 0.0045 | *COL1A1, CSNK1G2, DAB2, USP8, SFRP4* |
| GO Process | GO:0070887 | Cellular response to chemical stimulus | 0.6 | 0.0045 | *PLAT, COL1A1, STAT3, HPX, SMAD1, CASP3, DAB2, STAT1, DDR2, POSTN, USP8, TF* |
| GO Process | GO:0051171 | Regulation of nitrogen compound metabolic process | 0.4 | 0.0049 | *PLAT, COL1A1, STAT3, HPX, MAP2K1, SMAD1, CASP3, DAB2, STAT1, DDR2, YWHAZ, USP8, TF, PRKCA, SFRP4, SERPINH1, KNG1* |
| GO Process | GO:0008285 | Negative regulation of cell population proliferation | 0.92 | 0.0051 | *STAT3, MAP2K1, SMAD1, CASP3, DAB2, STAT1, SFRP4* |
| GO Process | GO:0006468 | Protein phosphorylation | 0.91 | 0.0057 | *CSNK1G2, HPX, MAP2K1, SMAD1, DDR2, YWHAZ, PRKCA* |
| GO Process | GO:0009653 | Anatomical structure morphogenesis | 0.63 | 0.0057 | *COL1A1, STAT3, MAP2K1, SMAD1, CASP3, YWHAZ, ACAN, PRKCA, SFRP4, SERPINH1, CSMD1* |
| GO Process | GO:0030198 | Extracellular matrix organization | 1.19 | 0.0057 | *COL1A1, DDR2, POSTN, ACAN, SERPINH1* |
| GO Process | GO:0042592 | Homeostatic process | 0.74 | 0.0057 | *STAT3, HPX, SMAD1, CASP3, STAT1, LCN2, TF, SFRP4, CSMD1* |
| GO Process | GO:0048513 | Animal organ development | 0.54 | 0.0057 | *COL1A1, STAT3, MAP2K1, SMAD1, CASP3, STAT1, DDR2, YWHAZ, TF, ACAN, SFRP4, SERPINH1, CSMD1* |
| GO Process | GO:0048545 | Response to steroid hormone | 1.18 | 0.0057 | *PLAT, COL1A1, CASP3, DAB2, USP8* |
| GO Process | GO:0048731 | System development | 0.49 | 0.0057 | *COL1A1, STAT3, MAP2K1, SMAD1, CASP3, STAT1, DDR2, YWHAZ, TF, ACAN, PRKCA, SFRP4, SERPINH1, CSMD1* |
| GO Process | GO:0048856 | Anatomical structure development | 0.43 | 0.0057 | *COL1A1, STAT3, MAP2K1, SMAD1, CASP3, DAB2, STAT1, DDR2, POSTN, YWHAZ, TF, ACAN, PRKCA, SFRP4, SERPINH1, CSMD1* |
| GO Process | GO:0080090 | Regulation of primary metabolic process | 0.39 | 0.0057 | *PLAT, COL1A1, STAT3, HPX, MAP2K1, SMAD1, CASP3, DAB2, STAT1, DDR2, YWHAZ, USP8, TF, PRKCA, SFRP4, SERPINH1, KNG1* |
| GO Process | GO:0071407 | Cellular response to organic cyclic compound | 1.01 | 0.0065 | *PLAT, COL1A1, SMAD1, CASP3, STAT1, USP8* |
| GO Process | GO:0001501 | Skeletal system development | 1 | 0.0067 | *COL1A1, SMAD1, DDR2, ACAN, SFRP4, SERPINH1* |
| GO Process | GO:0019222 | Regulation of metabolic process | 0.36 | 0.0067 | *PLAT, COL1A1, STAT3, HPX, MAP2K1, SMAD1, CASP3, DAB2, STAT1, DDR2, LCN2, YWHAZ, USP8, TF, PRKCA, SFRP4, SERPINH1, KNG1* |
| GO Process | GO:0070372 | Regulation of ERK1 and ERK2 cascade | 1.16 | 0.0067 | *MAP2K1, DAB2, DDR2, YWHAZ, PRKCA* |
| GO Process | GO:0009605 | Response to external stimulus | 0.6 | 0.0070 | *COL1A1, HPX, MAP2K1, CASP3, STAT1, DDR2, LCN2, POSTN, TF, CSMD1, KNG1* |
| GO Process | GO:0051240 | Positive regulation of multicellular organismal process | 0.71 | 0.0077 | *PLAT, COL1A1, STAT3, MAP2K1, SMAD1, DAB2, STAT1, LCN2, PRKCA* |
| GO Process | GO:0007259 | Receptor signaling pathway via JAK-STAT | 1.7 | 0.0091 | *STAT3, HPX, STAT1* |
| GO Process | GO:2000147 | Positive regulation of cell motility | 0.97 | 0.0092 | *COL1A1, STAT3, DAB2, DDR2, TF, PRKCA* |
| GO Process | GO:0006826 | Iron ion transport | 1.69 | 0.0095 | *HPX, LCN2, TF* |
| GO Process | GO:0048518 | Positive regulation of biological process | 0.37 | 0.0095 | *PLAT, COL1A1, CSNK1G2, STAT3, HPX, MAP2K1, SMAD1, CASP3, DAB2, STAT1, DDR2, LCN2, USP8, TF, PRKCA, SFRP4, KNG1* |
| GO Process | GO:1903034 | Regulation of response to wounding | 1.33 | 0.0095 | *PLAT, MAP2K1, DDR2, KNG1* |
| GO Process | GO:0031960 | Response to corticosteroid | 1.31 | 0.0098 | *PLAT, COL1A1, CASP3, USP8* |
| GO Process | GO:0048260 | Positive regulation of receptor-mediated endocytosis | 1.67 | 0.0098 | *DAB2, TF, SFRP4* |
| GO Process | GO:0051216 | Cartilage development | 1.31 | 0.0098 | *COL1A1, SMAD1, ACAN, SERPINH1* |
| GO Process | GO:0060255 | Regulation of macromolecule metabolic process | 0.37 | 0.0098 | *PLAT, COL1A1, STAT3, HPX, MAP2K1, SMAD1, CASP3, DAB2, STAT1, DDR2, YWHAZ, USP8, TF, PRKCA, SFRP4, SERPINH1, KNG1* |
| GO Process | GO:0070371 | ERK1 and ERK2 cascade | 1.67 | 0.0098 | *MAP2K1, YWHAZ, TF* |
| GO Process | GO:0071310 | Cellular response to organic substance | 0.63 | 0.0098 | *PLAT, COL1A1, STAT3, HPX, SMAD1, CASP3, DAB2, STAT1, DDR2, USP8* |
| GO Process | GO:0071417 | Cellular response to organonitrogen compound | 0.95 | 0.0098 | *PLAT, COL1A1, STAT3, CASP3, STAT1, DDR2* |
| GO Process | GO:0035295 | Tube development | 0.83 | 0.0100 | *MAP2K1, SMAD1, CASP3, STAT1, YWHAZ, PRKCA, CSMD1* |
| GO Process | GO:0043434 | Response to peptide hormone | 1.08 | 0.0106 | *PLAT, COL1A1, STAT3, STAT1, DDR2* |
| GO Process | GO:0051716 | Cellular response to stimulus | 0.36 | 0.0107 | *PLAT, COL1A1, CSNK1G2, STAT3, HPX, MAP2K1, SMAD1, CASP3, DAB2, STAT1, DDR2, POSTN, YWHAZ, USP8, TF, PRKCA, SFRP4* |
| GO Process | GO:0072359 | Circulatory system development | 0.82 | 0.0112 | *COL1A1, MAP2K1, SMAD1, CASP3, YWHAZ, ACAN, PRKCA* |
| GO Process | GO:0048878 | Chemical homeostasis | 0.82 | 0.0113 | *STAT3, HPX, STAT1, LCN2, TF, SFRP4, CSMD1* |
| GO Process | GO:0006879 | Cellular iron ion homeostasis | 1.61 | 0.0118 | *HPX, LCN2, TF* |
| GO Process | GO:0042127 | Regulation of cell population proliferation | 0.66 | 0.0120 | *STAT3, MAP2K1, SMAD1, CASP3, DAB2, STAT1, DDR2, PRKCA, SFRP4* |
| GO Process | GO:0032964 | Collagen biosynthetic process | 2.23 | 0.0161 | *COL1A1, SERPINH1* |
| GO Process | GO:0034103 | Regulation of tissue remodeling | 1.53 | 0.0186 | *DDR2, TF, PRKCA* |
| GO Process | GO:0009887 | Animal organ morphogenesis | 0.78 | 0.0191 | *COL1A1, STAT3, MAP2K1, ACAN, SFRP4, SERPINH1, CSMD1* |
| GO Process | GO:0000165 | MAPK cascade | 1.19 | 0.0214 | *MAP2K1, SMAD1, YWHAZ, TF* |
| GO Process | GO:0006952 | Defense response | 0.69 | 0.0214 | *STAT3, HPX, SMAD1, AOC3, STAT1, LCN2, TF, KNG1* |
| GO Process | GO:0007169 | Transmembrane receptor protein tyrosine kinase signaling pathway | 1 | 0.0214 | *PLAT, COL1A1, STAT3, CASP3, DDR2* |
| GO Process | GO:0038063 | Collagen-activated tyrosine kinase receptor signaling pathway | 2.15 | 0.0214 | *COL1A1, DDR2* |
| GO Process | GO:0060333 | Interferon-gamma-mediated signaling pathway | 2.15 | 0.0214 | *HPX, STAT1* |
| GO Process | GO:0048705 | Skeletal system morphogenesis | 1.17 | 0.0238 | *COL1A1, ACAN, SFRP4, SERPINH1* |
| GO Process | GO:1901701 | Cellular response to oxygen-containing compound | 0.75 | 0.0246 | *PLAT, COL1A1, STAT3, STAT1, DDR2, POSTN, USP8* |
| GO Process | GO:1901678 | Iron coordination entity transport | 2.09 | 0.0253 | *HPX, LCN2* |
| GO Process | GO:0042981 | Regulation of apoptotic process | 0.67 | 0.0269 | *CASP3, DAB2, STAT1, DDR2, YWHAZ, PRKCA, SFRP4, KNG1* |
| GO Process | GO:0071375 | Cellular response to peptide hormone stimulus | 1.15 | 0.0269 | *PLAT, STAT3, STAT1, DDR2* |
| GO Process | GO:0051591 | Response to cAMP | 1.45 | 0.0275 | *PLAT, COL1A1, STAT1* |
| GO Process | GO:0030154 | Cell differentiation | 0.47 | 0.0303 | *COL1A1, STAT3, MAP2K1, SMAD1, CASP3, DAB2, STAT1, YWHAZ, TF, ACAN, SFRP4, SERPINH1* |
| GO Process | GO:0032870 | Cellular response to hormone stimulus | 0.94 | 0.0335 | *PLAT, STAT3, STAT1, DDR2, USP8* |
| GO Process | GO:0045639 | Positive regulation of myeloid cell differentiation | 1.41 | 0.0336 | *STAT3, STAT1, PRKCA* |
| GO Process | GO:0060349 | Bone morphogenesis | 1.41 | 0.0336 | *COL1A1, SFRP4, SERPINH1* |
| GO Process | GO:0044092 | Negative regulation of molecular function | 0.72 | 0.0347 | *CASP3, AOC3, DAB2, DDR2, SFRP4, SERPINH1, KNG1* |
| GO Process | GO:0042542 | Response to hydrogen peroxide | 1.4 | 0.0348 | *COL1A1, CASP3, STAT1* |
| GO Process | GO:0050793 | Regulation of developmental process | 0.54 | 0.0348 | *COL1A1, STAT3, MAP2K1, SMAD1, DAB2, STAT1, DDR2, YWHAZ, PRKCA, SFRP4* |
| GO Process | GO:1901655 | Cellular response to ketone | 1.4 | 0.0348 | *PLAT, POSTN, USP8* |
| GO Process | GO:1901700 | Response to oxygen-containing compound | 0.65 | 0.0348 | *PLAT, COL1A1, STAT3, CASP3, STAT1, DDR2, POSTN, USP8* |
| GO Process | GO:2000641 | Regulation of early endosome to late endosome transport | 1.98 | 0.0348 | *MAP2K1, DAB2* |
| GO Process | GO:0034097 | Response to cytokine | 0.81 | 0.0355 | *COL1A1, STAT3, HPX, CASP3, STAT1, PRKCA* |
| GO Process | GO:0048522 | Positive regulation of cellular process | 0.36 | 0.0355 | *COL1A1, CSNK1G2, STAT3, HPX, MAP2K1, SMAD1, CASP3, DAB2, STAT1, DDR2, USP8, TF, PRKCA, SFRP4, KNG1* |
| GO Process | GO:0045780 | Positive regulation of bone resorption | 1.95 | 0.0361 | *TF, PRKCA* |
| GO Process | GO:0033993 | Response to lipid | 0.79 | 0.0397 | *PLAT, COL1A1, STAT3, CASP3, DAB2, USP8* |
| GO Process | GO:0030335 | Positive regulation of cell migration | 0.91 | 0.0416 | *COL1A1, STAT3, DAB2, DDR2, PRKCA* |
| GO Process | GO:0045765 | Regulation of angiogenesis | 1.08 | 0.0429 | *STAT3, SMAD1, STAT1, PRKCA* |
| GO Process | GO:0065007 | Biological regulation | 0.18 | 0.0433 | *PLAT, COL1A1, CSNK1G2, STAT3, HPX, MAP2K1, SMAD1, CASP3, AOC3, DAB2, STAT1, DDR2, LCN2, POSTN, YWHAZ, USP8, TF, PRKCA, SFRP4, SERPINH1, CSMD1, KNG1* |
| GO Process | GO:0071295 | Cellular response to vitamin | 1.89 | 0.0445 | *COL1A1, POSTN* |
| GO Process | GO:0032501 | Multicellular organismal process | 0.32 | 0.0448 | *PLAT, COL1A1, STAT3, MAP2K1, SMAD1, CASP3, STAT1, DDR2, YWHAZ, TF, ACAN, PRKCA, SFRP4, SERPINH1, CSMD1, KNG1* |
| GO Process | GO:0051246 | Regulation of protein metabolic process | 0.51 | 0.0472 | *PLAT, STAT3, HPX, MAP2K1, CASP3, DAB2, DDR2, USP8, SERPINH1, KNG1* |
| GO Process | GO:0032355 | Response to estradiol | 1.33 | 0.0476 | *COL1A1, STAT3, CASP3* |
| GO Process | GO:0042325 | Regulation of phosphorylation | 0.68 | 0.0498 | *STAT3, HPX, MAP2K1, CASP3, DAB2, DDR2, TF* |
| GO Process | GO:2000637 | Positive regulation of miRNA-mediated gene silencing | 1.85 | 0.0498 | *STAT3, MAP2K1* |
| GO Component | GO:0062023 | Collagen-containing extracellular matrix | 1.1 | 0.0124 | *COL1A1, HPX, POSTN, ACAN, SERPINH1, KNG1* |
| GO Component | GO:0072562 | Blood microparticle | 1.46 | 0.0124 | *HPX, YWHAZ, TF, KNG1* |
| GO Component | GO:0031410 | Cytoplasmic vesicle | 0.58 | 0.0228 | *PLAT, COL1A1, HPX, MAP2K1, AOC3, DAB2, LCN2, YWHAZ, USP8, TF, KNG1* |
| GO Component | GO:0071944 | Cell periphery | 0.36 | 0.0391 | *COL1A1, CSNK1G2, STAT3, HPX, MAP2K1, CASP3, AOC3, DAB2, DDR2, POSTN, USP8, TF, ACAN, PRKCA, SERPINH1, KNG1* |
| GO Component | GO:0012505 | Endomembrane system | 0.4 | 0.0453 | *PLAT, COL1A1, MAP2K1, SMAD1, AOC3, DAB2, LCN2, POSTN, USP8, TF, ACAN, PRKCA, SERPINH1, KNG1* |
| KEGG | hsa05161 | Hepatitis B | 1.51 | 9.10E-06 | *STAT3, MAP2K1, CASP3, STAT1, YWHAZ, PRKCA* |
| KEGG | hsa04933 | AGE-RAGE signaling pathway in diabetic complications | 1.65 | 1.67E-05 | *COL1A1, STAT3, CASP3, STAT1, PRKCA* |
| KEGG | hsa05160 | Hepatitis C | 1.44 | 0.0001 | *STAT3, MAP2K1, CASP3, STAT1, YWHAZ* |
| KEGG | hsa05205 | Proteoglycans in cancer | 1.34 | 0.0002 | *COL1A1, STAT3, MAP2K1, CASP3, PRKCA* |
| KEGG | hsa04066 | HIF-1 signaling pathway | 1.53 | 0.0004 | *STAT3, MAP2K1, TF, PRKCA* |
| KEGG | hsa04935 | Growth hormone synthesis, secretion and action | 1.47 | 0.0006 | *STAT3, MAP2K1, STAT1, PRKCA* |
| KEGG | hsa05200 | Pathways in cancer | 1 | 0.0011 | *STAT3, MAP2K1, CASP3, STAT1, PRKCA, KNG1* |
| KEGG | hsa05164 | Influenza A | 1.32 | 0.0015 | *MAP2K1, CASP3, STAT1, PRKCA* |
| KEGG | hsa05206 | MicroRNAs in cancer | 1.33 | 0.0015 | *STAT3, MAP2K1, CASP3, PRKCA* |
| KEGG | hsa05167 | Kaposi sarcoma-associated herpesvirus infection | 1.26 | 0.0022 | *STAT3, MAP2K1, CASP3, STAT1* |
| KEGG | hsa04917 | Prolactin signaling pathway | 1.58 | 0.0023 | *STAT3, MAP2K1, STAT1* |
| KEGG | hsa05212 | Pancreatic cancer | 1.56 | 0.0023 | *STAT3, MAP2K1, STAT1* |
| KEGG | hsa05223 | Non-small cell lung cancer | 1.58 | 0.0023 | *STAT3, MAP2K1, PRKCA* |
| KEGG | hsa01521 | EGFR tyrosine kinase inhibitor resistance | 1.52 | 0.0026 | *STAT3, MAP2K1, PRKCA* |
| KEGG | hsa05163 | Human cytomegalovirus infection | 1.2 | 0.0026 | *STAT3, MAP2K1, CASP3, PRKCA* |
| KEGG | hsa05235 | PD-L1 expression and PD-1 checkpoint pathway in cancer | 1.47 | 0.0032 | *STAT3, MAP2K1, STAT1* |
| KEGG | hsa05145 | Toxoplasmosis | 1.4 | 0.0046 | *STAT3, CASP3, STAT1* |
| KEGG | hsa05146 | Amoebiasis | 1.41 | 0.0046 | *COL1A1, CASP3, PRKCA* |
| KEGG | hsa04726 | Serotonergic synapse | 1.38 | 0.0050 | *MAP2K1, CASP3, PRKCA* |
| KEGG | hsa04071 | Sphingolipid signaling pathway | 1.35 | 0.0058 | *MAP2K1, PRKCA, KNG1* |
| KEGG | hsa04650 | Natural killer cell mediated cytotoxicity | 1.33 | 0.0061 | *MAP2K1, CASP3, PRKCA* |
| KEGG | hsa04919 | Thyroid hormone signaling pathway | 1.33 | 0.0061 | *MAP2K1, STAT1, PRKCA* |
| KEGG | hsa04926 | Relaxin signaling pathway | 1.31 | 0.0064 | *COL1A1, MAP2K1, PRKCA* |
| KEGG | hsa05165 | Human papillomavirus infection | 1.02 | 0.0073 | *COL1A1, MAP2K1, CASP3, STAT1* |
| KEGG | hsa05162 | Measles | 1.27 | 0.0075 | *STAT3, CASP3, STAT1* |
| KEGG | hsa04550 | Signaling pathways regulating pluripotency of stem cells | 1.26 | 0.0078 | *STAT3, MAP2K1, SMAD1* |
| KEGG | hsa04151 | PI3K-Akt signaling pathway | 0.99 | 0.0085 | *COL1A1, MAP2K1, YWHAZ, PRKCA* |
| KEGG | hsa05143 | African trypanosomiasis | 1.68 | 0.0107 | *PRKCA, KNG1* |
| KEGG | hsa05203 | Viral carcinogenesis | 1.15 | 0.0147 | *STAT3, CASP3, YWHAZ* |
| KEGG | hsa04062 | Chemokine signaling pathway | 1.14 | 0.0149 | *STAT3, MAP2K1, STAT1* |
| KEGG | hsa05169 | Epstein-Barr virus infection | 1.13 | 0.0158 | *STAT3, CASP3, STAT1* |
| KEGG | hsa04510 | Focal adhesion | 1.12 | 0.0160 | *COL1A1, MAP2K1, PRKCA* |
| KEGG | hsa05170 | Human immunodeficiency virus 1 infection | 1.1 | 0.0174 | *MAP2K1, CASP3, PRKCA* |
| KEGG | hsa04370 | VEGF signaling pathway | 1.49 | 0.0204 | *MAP2K1, PRKCA* |
| KEGG | hsa04730 | Long-term depression | 1.46 | 0.0219 | *MAP2K1, PRKCA* |
| KEGG | hsa05321 | Inflammatory bowel disease | 1.46 | 0.0219 | *STAT3, STAT1* |
| KEGG | hsa04720 | Long-term potentiation | 1.43 | 0.0235 | *MAP2K1, PRKCA* |
| KEGG | hsa04929 | GnRH secretion | 1.43 | 0.0235 | *MAP2K1, PRKCA* |
| KEGG | hsa04664 | Fc epsilon RI signaling pathway | 1.42 | 0.0237 | *MAP2K1, PRKCA* |
| KEGG | hsa05221 | Acute myeloid leukemia | 1.41 | 0.0244 | *STAT3, MAP2K1* |
| KEGG | hsa05214 | Glioma | 1.38 | 0.0266 | *MAP2K1, PRKCA* |
| KEGG | hsa04010 | MAPK signaling pathway | 0.95 | 0.0334 | *MAP2K1, CASP3, PRKCA* |
| KEGG | hsa04012 | ErbB signaling pathway | 1.33 | 0.0334 | *MAP2K1, PRKCA* |
| KEGG | hsa04610 | Complement and coagulation cascades | 1.32 | 0.0334 | *PLAT, KNG1* |
| KEGG | hsa05210 | Colorectal cancer | 1.32 | 0.0334 | *MAP2K1, CASP3* |
| KEGG | hsa04540 | Gap junction | 1.29 | 0.0350 | *MAP2K1, PRKCA* |
| KEGG | hsa04912 | GnRH signaling pathway | 1.29 | 0.0350 | *MAP2K1, PRKCA* |
| KEGG | hsa04657 | IL-17 signaling pathway | 1.27 | 0.0358 | *CASP3, LCN2* |
| KEGG | hsa04666 | Fc gamma R-mediated phagocytosis | 1.28 | 0.0358 | *MAP2K1, PRKCA* |
| KEGG | hsa04750 | Inflammatory mediator regulation of TRP channels | 1.27 | 0.0358 | *PRKCA, KNG1* |
| KEGG | hsa04916 | Melanogenesis | 1.26 | 0.0373 | *MAP2K1, PRKCA* |
| KEGG | hsa05231 | Choline metabolism in cancer | 1.26 | 0.0373 | *MAP2K1, PRKCA* |
| KEGG | hsa05215 | Prostate cancer | 1.25 | 0.0374 | *PLAT, MAP2K1* |
| KEGG | hsa04620 | Toll-like receptor signaling pathway | 1.23 | 0.0381 | *MAP2K1, STAT1* |
| KEGG | hsa04659 | Th17 cell differentiation | 1.24 | 0.0381 | *STAT3, STAT1* |
| KEGG | hsa04928 | Parathyroid hormone synthesis, secretion and action | 1.22 | 0.0404 | *MAP2K1, PRKCA* |
| KEGG | hsa04725 | Cholinergic synapse | 1.2 | 0.0433 | *MAP2K1, PRKCA* |
| KEGG | hsa04668 | TNF signaling pathway | 1.19 | 0.0441 | *MAP2K1, CASP3* |
| Reactome | HSA-1474244 | Extracellular matrix organization | 1.23 | 0.0025 | *COL1A1, CASP3, DDR2, ACAN, PRKCA, SERPINH1* |
| Reactome | HSA-9006934 | Signaling by Receptor Tyrosine Kinases | 1.06 | 0.0025 | *PLAT, COL1A1, STAT3, MAP2K1, STAT1, USP8, PRKCA* |
| Reactome | HSA-162582 | Signal Transduction | 0.61 | 0.0055 | *PLAT, COL1A1, CSNK1G2, STAT3, MAP2K1, SMAD1, CASP3, STAT1, YWHAZ, USP8, PRKCA, KNG1* |
| Reactome | HSA-449147 | Signaling by Interleukins | 1.05 | 0.0063 | *STAT3, MAP2K1, CASP3, STAT1, LCN2, YWHAZ* |
| Reactome | HSA-76002 | Platelet activation, signaling and aggregation | 1.22 | 0.0063 | *COL1A1, YWHAZ, TF, PRKCA, KNG1* |
| Reactome | HSA-1433557 | Signaling by SCF-KIT | 1.78 | 0.0077 | *STAT3, STAT1, PRKCA* |
| Reactome | HSA-109606 | Intrinsic Pathway for Apoptosis | 1.69 | 0.0114 | *STAT3, CASP3, YWHAZ* |
| Reactome | HSA-109582 | Hemostasis | 0.93 | 0.0136 | *PLAT, COL1A1, YWHAZ, TF, PRKCA, KNG1* |
| Reactome | HSA-186797 | Signaling by PDGF | 1.65 | 0.0136 | *PLAT, STAT3, STAT1* |
| Reactome | HSA-3000171 | Non-integrin membrane-ECM interactions | 1.64 | 0.0136 | *COL1A1, DDR2, PRKCA* |
| Reactome | HSA-8985947 | Interleukin-9 signaling | 2.28 | 0.0148 | *STAT3, STAT1* |
| Reactome | HSA-9020958 | Interleukin-21 signaling | 2.23 | 0.0162 | *STAT3, STAT1* |
| Reactome | HSA-1059683 | Interleukin-6 signaling | 2.19 | 0.0177 | *STAT3, STAT1* |
| Reactome | HSA-430116 | GP1b-IX-V activation signaling | 2.15 | 0.0177 | *COL1A1, YWHAZ* |
| Reactome | HSA-6806834 | Signaling by MET | 1.51 | 0.0177 | *COL1A1, STAT3, USP8* |
| Reactome | HSA-8984722 | Interleukin-35 Signaling | 2.15 | 0.0177 | *STAT3, STAT1* |
| Reactome | HSA-9020956 | Interleukin-27 signaling | 2.19 | 0.0177 | *STAT3, STAT1* |
| Reactome | HSA-9673767 | Signaling by PDGFRA transmembrane, juxtamembrane and kinase domain mutants | 2.15 | 0.0177 | *STAT3, STAT1* |
| Reactome | HSA-9673770 | Signaling by PDGFRA extracellular domain mutants | 2.15 | 0.0177 | *STAT3, STAT1* |
| Reactome | HSA-1839117 | Signaling by cytosolic FGFR1 fusion mutants | 1.98 | 0.0265 | *STAT3, STAT1* |
| Reactome | HSA-6785807 | Interleukin-4 and Interleukin-13 signaling | 1.38 | 0.0286 | *STAT3, STAT1, LCN2* |
| Reactome | HSA-9670439 | Signaling by phosphorylated juxtamembrane, extracellular and kinase domain KIT mutants | 1.93 | 0.0294 | *STAT3, STAT1* |
| Reactome | HSA-195721 | Signaling by WNT | 1.06 | 0.0328 | *CSNK1G2, YWHAZ, USP8, PRKCA* |
| Reactome | HSA-8878166 | Transcriptional regulation by RUNX2 | 1.33 | 0.0328 | *COL1A1, SMAD1, STAT1* |
| Reactome | HSA-9705462 | Inactivation of CSF3 (G-CSF) signaling | 1.85 | 0.0328 | *STAT3, STAT1* |
| Reactome | HSA-982772 | Growth hormone receptor signaling | 1.87 | 0.0328 | *STAT3, STAT1* |
| Reactome | HSA-8854691 | Interleukin-20 family signaling | 1.84 | 0.0330 | *STAT3, STAT1* |
| Reactome | HSA-76005 | Response to elevated platelet cytosolic Ca2+ | 1.29 | 0.0351 | *TF, PRKCA, KNG1* |
| Reactome | HSA-3000170 | Syndecan interactions | 1.8 | 0.0358 | *COL1A1, PRKCA* |
| Reactome | HSA-1474228 | Degradation of the extracellular matrix | 1.26 | 0.0398 | *COL1A1, CASP3, ACAN* |
| Reactome | HSA-186763 | Downstream signal transduction | 1.77 | 0.0398 | *STAT3, STAT1* |
| Reactome | HSA-8941326 | RUNX2 regulates bone development | 1.74 | 0.0415 | *COL1A1, SMAD1* |
| Reactome | HSA-9680350 | Signaling by CSF1 (M-CSF) in myeloid cells | 1.74 | 0.0415 | *STAT3, STAT1* |

**S5 Table. Functionally enriched pathways derived from the 23 differentially expressed proteins in the hypothesis-based analysis**. ID: Enriched category name. STR: Strength of protein-protein interaction (PPI). FDR: False Discovery Rate (Adjusted p-value). Overall PPI enrichment p-value: 1.35E-07. Hypothesis-based candidate protein preselection may influence enrichment results. Figure 6 summarizes enriched pathways for individual genes.
