# Supplementary material for: Identification of novel plasma proteomic biomarkers of Dupuytren disease
Source: PLoS One. 2026 Mar 18;21(3):e0343733. doi: 10.1371/journal.pone.0343733 (PMC12998848; doi:10.1371/journal.pone.0343733)
Supplement: S6 Table — This table lists all gene names referenced in the manuscript, their UniProt identifiers, protein names, and gene aliases. (DOCX) [file pone.0343733.s011.docx]

| **Gene** | **UniProt** | **Proteins and gene aliases (Separated by ";" Character)** |
| --- | --- | --- |
| *ACAN* | P16112 | *ACAN*; aggrecan; CSPGCP; aggrecan proteoglycan; chondroitin sulfate proteoglycan 1; aggrecan 1; AGC1; AGCAN; CSPG1; SEDK; SSOAOD; cartilage-specific proteoglycan core protein; chondroitin sulfate proteoglycan core protein 1; large aggregating proteoglycan; Aggrecan core protein; CSPCP |
| *AKR1A1* | P14550 | *AKR1A1*; aldo-keto reductase family 1 member A1; ALR; DD3; dihydrodiol dehydrogenase 3; aldo-keto reductase family 1, member A1 (aldehyde reductase); HEL-S-6; HEL-S-165mP; epididymis secretory protein Li 6; epididymis secretory sperm binding protein Li 165mP; glucuronate reductase; glucuronolactone reductase; Alcohol dehydrogenase [NADP(+)] |
| *AOC3* | Q16853 | *AOC3*; amine oxidase copper containing 3; VAP1; HPAO; VAP-1; vascular adhesion protein 1; SSAO; membrane primary amine oxidase; amine oxidase, copper containing 3 (vascular adhesion protein 1); copper amine oxidase; placenta copper monamine oxidase; semicarbazide-sensitive amine oxidase |
| *ARG2* | P78540 | *ARG2*; Arginase 2; Arginase-2 Mitochondrial; Kidney-Type Arginase; Non-Hepatic Arginase; Arginase Type II; Type II Arginase; Arginase II; EC 3.5.3.1; L-Arginine Amidinohydrolase; L-Arginine Ureahydrolase; Nonhepatic Arginase; Kidney Arginase; EC 3.5.3 |
| *BIN2* | Q9UBW5 | *BIN2*; Bridging Integrator 2; BRAP-1; Breast Cancer-Associated Protein 1; Breast Cancer Associated Protein BRAP1; BRAP1 |
| *C5* | P01031 | *C5*; complement C5; CPAMD4; C5a; C5b; prepro-C5; C5a anaphylatoxin; complement component 5; C5D; ECLZB; C3 and PZP-like alpha-2-macroglobulin domain-containing protein 4; anaphylatoxin C5a analog |
| *C6* | P13671 | *C6*; complement C6; complement component 6; complement component C6 |
| *CALCB* | P10092 | *CALCB*; Calcitonin Related Polypeptide Beta; CGRP-II; CALC2; Calcitonin Gene-Related Peptide II; Calcitonin Gene-Related Peptide 2; Beta-Type CGRP; Calcitonin 2; Beta-CGRP; FLJ30166; CGRP2 |
| *CASP3* | P42574 | *CASP3*; caspase 3; CPP32; CPP32B; Yama; apopain; caspase 3, apoptosis-related cysteine protease; caspase 3, apoptosis-related cysteine peptidase; SCA-1; caspase-3; CASP-3; CPP-32; PARP cleavage protease; SREBP cleavage activity 1; cysteine protease CPP32; procaspase3; protein Yama |
| *CGA* | P01215 | *CGA*; glycoprotein hormones, alpha polypeptide; HCG; GPHa; GPHA1; FSHA; LHA; TSHA; GPA1; follicle-stimulating hormone alpha subunit; chorionic gonadotropin, alpha polypeptide; luteinizing hormone alpha chain; lutropin alpha chain; thyroid-stimulating hormone alpha chain; glycoprotein hormones alpha chain; CG-ALPHA; FSH-alpha; LSH-alpha; TSH-alpha; anterior pituitary glycoprotein hormones common subunit alpha; choriogonadotropin alpha chain; chorionic gonadotrophin subunit alpha; follicle-stimulating hormone alpha chain; follitropin alpha chain; thyrotropin alpha chain |
| *CGB3* | P0DN86 | *CGB3*; Chorionic Gonadotropin Subunit Beta 3; CGB; Chorionic Gonadotropin Beta Polypeptide; Chorionic Gonadotropin Beta Subunit 3; Choriogonadotropin Subunit Beta 3; Chorionic Gonadotropin Chain Beta; CG-Beta; Chorionic Gonadotropin Beta 3 Subunit; Chorionic Gonadotrophin Chain Beta; Chorionic Gonadotropin Beta Chain; Luteinizing Hormone Beta Subunit; Choriogonadotropin Subunit Beta; CGB5; CGB7; CGB8; HCGB; LHB |
| *CGB7* | P0DN87 | *CGB7*; Chorionic Gonadotropin Subunit Beta 7; CG-Beta-A; Chorionic Gonadotropin Beta Polypeptide 7; Chorionic Gonadotropin Beta Subunit 7; Choriogonadotropin Subunit Beta 7; Chorionic Gonadotropin Beta 7 Subunit; CGB6 |
| *COL1A1* | P02452 | *COL1A1*; collagen type I alpha 1 chain; collagen, type I, alpha 1; CAFYD; EDSARTH1; OI1; OI2; OI3; collagen alpha-1(I) chain; alpha1(I) procollagen; collagen alpha 1 chain type I; collagen alpha-1(I) chain preproprotein; collagen of skin, tendon and bone, alpha-1 chain; pro-alpha-1 collagen type 1; type I proalpha 1; type I procollagen alpha 1 chain; Alpha-1 type I collagen |
| *CPB1* | P15086 | *CPB1*; Carboxypeptidase B1; Pancreatic Carboxypeptidase B; Carboxypeptidase B1 (Tissue); Tissue Carboxypeptidase B; Pancreas-Specific Protein; Carboxypeptidase B; Protaminase; EC 3.4.17.2; PASP; PCPB; CPB; Procarboxypeptidase B; EC 3.4.17 |
| *CRKL* | P46109 | *CRKL*; CRK Like Proto-Oncogene; V-Crk Avian Sarcoma Virus CT10 Oncogene Homolog-Like; Crk-Like Protein |
| *CSMD1* | Q96PZ7 | *CSMD1*; CUB and Sushi multiple domains 1; KIAA1890; PPP1R24; protein phosphatase 1, regulatory subunit 24; CUB and sushi domain-containing protein 1; CUB and sushi multiple domains protein 1 |
| *CSNK1G2* | P78368 | *CSNK1G2*; Casein Kinase 1 Gamma 2; CK1g2; Casein Kinase I Isoform Gamma-2; CKI-Gamma 2; Casein Kinase 1 Gamma 2; Casein Kinase I; CK1G2 |
| *DAB2* | P98082 | *DAB2*; DAB adaptor protein 2; DOC-2; disabled (Drosophila) homolog 2 (mitogen-responsive phosphoprotein); disabled homolog 2, mitogen-responsive phosphoprotein (Drosophila); Dab, mitogen-responsive phosphoprotein, homolog 2 (Drosophila); DAB2, clathrin adaptor protein; DOC2; disabled homolog 2; Dab, mitogen-responsive phosphoprotein, homolog 2; adaptor molecule disabled-2; differentially expressed in ovarian carcinoma 2; differentially-expressed protein 2; disabled homolog 2, mitogen-responsive phosphoprotein |
| *DDR2* | Q16832 | *DDR2*; discoidin domain receptor tyrosine kinase 2; TKT; discoidin domain receptor family, member 2; MIG20a; NTRKR3; TYRO10; WRCN; discoidin domain-containing receptor 2; CD167 antigen-like family member B; cell migration-inducing protein 20; discoidin domain receptor 2; discoidin domain-containing receptor tyrosine kinase 2; migration-inducing gene 16 protein; neurotrophic tyrosine kinase receptor related 3; receptor protein-tyrosine kinase TKT; tyrosine-protein kinase TYRO10; Neurotrophic tyrosine kinase, receptor-related 3; CD167b antigen |
| *DDX19A* | Q9NUU7 | *DDX19A*; DEAD-Box Helicase 19A; DDX19L; DEAD (Asp-Glu-Ala-Asp) Box Polypeptide 19A; ATP-Dependent RNA Helicase DDX19A; DEAD Box Protein 19A; DDX19-Like Protein; FLJ11126; DEAD (Asp-Glu-Ala-As) Box Polypeptide 19-Like; DEAD (Asp-Glu-Ala-As) Box Polypeptide 19A; DDX19-DDX19L; RNA Helicase; EC 3.6.4.13; EC 3.6.1 |
| *DSG2* | Q14126 | *DSG2*; Desmoglein 2; CDHF5; Cadherin Family Member 5; Desmoglein-2; HDGC |
| *EDIL3* | O43854 | *EDIL3*; EGF Like Repeats And Discoidin Domains 3; DEL1; EGF-Like Repeat And Discoidin I-Like Domain-Containing Protein 3; Developmentally-Regulated Endothelial Cell Locus 1 Protein; EGF-Like Repeats And Discoidin I-Like Domains 3; Integrin-Binding Protein DEL1; Developmental Endothelial Locus-1 |
| *EIF4H* | Q15056 | *EIF4H*; Eukaryotic Translation Initiation Factor 4H; WSCR1; KIAA0038; WBSCR1; Williams-Beuren Syndrome Chromosome Region 1; EIF-4H; Williams-Beuren Syndrome Chromosomal Region 1 Protein |
| *ELAPOR1* | Q6UXG2 | *ELAPOR1*; Endosome-Lysosome Associated Apoptosis And Autophagy Regulator 1; KIAA1324; EIG121; Endosome/Lysosome-Associated Apoptosis And Autophagy Regulator 1; Estrogen-Induced Gene 121 Protein; Maba1; Estrogen Induced Gene 121; MABA1 |
| *FAM234B* | A2RU67 | *FAM234B*; Family With Sequence Similarity 234 Member B; KIAA1467; Protein FAM234B |
| *FKBP5* | Q13451 | *FKBP5*; FKBP Prolyl Isomerase 5; FKBP51; FKBP54; P54; 54 KDa Progesterone Receptor-Associated Immunophilin; Peptidyl-Prolyl Cis-Trans Isomerase FKBP5; 51 KDa FK506-Binding Protein; Androgen-Regulated Protein 6; HSP90-Binding Immunophilin; FK506-Binding Protein 5; FK506 Binding Protein 5; PPIase FKBP5; 51 KDa FKBP; FF1 Antigen; EC 5.2.1.8; Rotamase; FKBP-51; PPIase; Ptg-10; AIG6; Peptidylprolyl Cis-Trans Isomerase; T-Cell FK506-Binding Protein; PPIASE; PTG-10; FKBP-5 |
| *G3BP2* | Q9UN86 | *G3BP2*; G3BP Stress Granule Assembly Factor 2; KIAA0660; Ras-GTPase Activating Protein SH3 Domain-Binding Protein 2; GTPase Activating Protein (SH3 Domain) Binding Protein 2; Ras GTPase-Activating Protein-Binding Protein 2; GAP SH3 Domain-Binding Protein 2; G3BP-2 |
| *GP1BB* | P13224 | *GP1BB*; Glycoprotein Ib Platelet Subunit Beta; Platelet Glycoprotein Ib Beta Chain; Glycoprotein Ib (Platelet) Beta Polypeptide; Antigen CD42b-Beta; GP-Ib Beta; GPIbbeta; CD42C; Nuclear Localization Signal Deleted In Velocardiofacial Syndrome; Platelet Membrane Glycoprotein Ib Beta; Glycoprotein Ib Platelet Beta Subunit; CD42c Antigen; GPIb-Beta; GPIBBETA; BDPLT1; CD42c; GPIBB; GPIbB; BS |
| *GUSB* | P08236 | *GUSB*; glucuronidase beta; glucuronidase, beta; BG; MPS7; beta-glucuronidase; beta-D-glucuronidase; beta-G1 |
| *HGS* | O14964 | *HGS*; Hepatocyte Growth Factor-Regulated Tyrosine Kinase Substrate; Protein Pp110; ZFYVE8; Human Growth Factor-Regulated Tyrosine Kinase Substrate; Vps27; VPS27 |
| *HPX* | P02790 | *HPX*; hemopexin; HX; beta-1B-glycoprotein |
| *HSP90AA1* | P07900 | *HSP90AA1*; Heat Shock Protein 90 Alpha Family Class A Member 1; HSP90N; HSPC1; HSPCA; Heat Shock Protein 90kDa Alpha (Cytosolic) Class A Member 1; Lipopolysaccharide-Associated Protein 2; Heat Shock 90kDa Protein 1 Alpha; Renal Carcinoma Antigen NY-REN-38; Heat Shock 90kD Protein 1 Alpha; Heat Shock Protein HSP 90-Alpha; LPS-Associated Protein 2; Heat Shock 86 KDa; FLJ31884; HSP90A; HSP 86; Hsp89; Hsp90; HSP86; LAP-2; Heat Shock Protein 90kDa Alpha Family Class A Member 1; Epididymis Secretory Sperm Binding Protein Li 65p; Epididymis Luminal Secretory Protein 52; Heat Shock 90kD Protein 1 Alpha-Like 4; Heat Shock 90kD Protein Alpha-Like 4; EC 3.6.4.10; HEL-S-65p; HSPCAL1; HSPCAL4; HSP89A; Hsp103; HSP89; HSP90; EL52; HSPN; LAP2 |
| *KNG1* | P01042 | *KNG1*; kininogen 1; BK; HMWK; alpha-2-thiol proteinase inhibitor; bradykinin; high-molecular-weight kininogen; kininogen; BDK; HAE6; HK; KNG; kininogen-1; Fitzgerald factor; high molecular weight kininogen; Williams-Fitzgerald-Flaujeac factor |
| *LCN2* | P80188 | *LCN2*; Lipocalin 2; NGAL; Neutrophil Gelatinase-Associated Lipocalin; Oncogene 24p3; 24p3; 25 KDa Alpha-2-Microglobulin-Related Subunit Of MMP-9; Siderocalin; P25; Migration-Stimulating Factor Inhibitor; Lipocalin 2 (Oncogene 24p3); Siderocalin LCN2; Lipocalin-2; MSFI; HNL |
| *MAP2K1* | Q02750 | *MAP2K1*; mitogen-activated protein kinase kinase 1; MEK1; MAPKK1; CFC3; MEL; MKK1; PRKMK1; dual specificity mitogen-activated protein kinase kinase 1; ERK activator kinase 1; MAPK/ERK kinase 1; MAPKK 1; MEK 1; protein kinase, mitogen-activated, kinase 1 (MAP kinase kinase 1); MAP kinase kinase 1 |
| *MAP3K11* | Q16584 | *MAP3K11*; Mitogen-Activated Protein Kinase Kinase Kinase 11; SPRK; MEKK11; MLK3; PTK1; Src-Homology 3 Domain-Containing Proline-Rich Kinase; Mixed Lineage Kinase 3; EC 2.7.11.25; SH3 Domain-Containing Proline-Rich Kinase; Protein-Tyrosine Kinase PTK1; EC 2.7.11; MLK-3 |
| *MBNL1* | Q9NR56 | *MBNL1*; Muscleblind-Like Protein 1; EXP42; EXP40; EXP35; Muscleblind-Like Splicing Regulator 1; Muscleblind (Drosophila)-Like; Muscleblind-Like (Drosophila); Muscleblind-Like |
| *MBNL2* | Q5VZF2 | *MBNL2*; Muscleblind Like Splicing Regulator 2; MBLL39; MBLL; Muscleblind-Like Protein-Like 39; Muscleblind-Like Protein 2; Muscleblind-Like Protein 1; Muscleblind-Like Splicing Regulator 2; Muscleblind-Like 2 (Drosophila); Muscleblind-Like Protein-Like; Muscleblind-Like 2; PRO2032; MLP1 |
| *NAA80* | Q93015 | *NAA80*; N-Alpha-Acetyltransferase 80; NatH Catalytic Subunit; FUS2; NAT6; N-Alpha-Acetyltransferase 80; N-Acetyltransferase 6; Protein Fusion-2; HsNAAA80; N(Alpha)-Acetyltransferase 80 NatH Catalytic Subunit; N-Acetyltransferase 6 (GCN5-Related); Protein Fus-2; EC 2.3.1.-; FUS-2 |
| *NRG4* | Q8WWG1 | *NRG4*; Neuregulin 4; HRG4; Pro-Neuregulin-4 Membrane-Bound Isoform; Pro-NRG4; Heregulin 4 |
| *OSCAR* | Q8IYS5 | *OSCAR*; Osteoclast Associated Ig-Like Receptor; Osteoclast Associated Immunoglobulin-Like Receptor; Osteoclast-Associated Immunoglobulin-Like Receptor; Polymeric Immunoglobulin Receptor 3; Poly-Ig Receptor 3; PIgR-3; Osteoclast Associated Receptor OSCAR-S1; Osteoclast Associated Receptor OSCAR-S2; Osteoclast-Associated Receptor; HOSCAR; PIGR3; PIgR3 |
| *PCSK9* | Q8NBP7 | *PCSK9*; proprotein convertase subtilisin/kexin type 9; NARC-1; FH3; hypercholesterolemia, autosomal dominant 3; FHCL3; HCHOLA3; LDLCQ1; NARC1; PC9; convertase subtilisin/kexin type 9 preproprotein; neural apoptosis regulated convertase 1; subtilisin/kexin-like protease PC9; Neural apoptosis-regulated convertase 1; Proprotein convertase 9 |
| *PLAT* | P00750 | *PLAT*; plasminogen activator, tissue type; plasminogen activator, tissue; T-PA; TPA; tissue-type plasminogen activator; plasminogen/activator kringle; reteplase; t-plasminogen activator |
| *POSTN* | Q15063 | *POSTN*; periostin; OSF-2; PN; osteoblast specific factor 2; periostin, osteoblast specific factor; OSF2; PDLPOSTN; osteoblast specific factor 2 (fasciclin I-like); periodontal ligament-specific periostin; Osteoblast-specific factor 2 |
| *PRKAR2B* | P31323 | *PRKAR2B*; Protein Kinase CAMP-Dependent Type II Regulatory Subunit Beta; Protein Kinase CAMP-Dependent Regulatory Subunit Type II Beta; CAMP-Dependent Protein Kinase Type II-Beta Regulatory Subunit; Protein Kinase CAMP-Dependent Regulatory Type II Beta; PRKAR2; CAMP-Dependent Protein Kinase Type II-Beta Regulatory Chain; WUGSC:H_RG363E19.2; H_RG363E19.2; RII-BETA |
| *PRKCA* | P17252 | *PRKCA*; protein kinase C alpha; PKC+¦; protein kinase C, alpha; AAG6; PKC-alpha; PKCA; PKCI+/-; PKCalpha; PRKACA; protein kinase C alpha type; PKC-A; aging-associated gene 6; PKC+Ä-¦ |
| *PRSS1* | P07477 | *PRSS1*; Serine Protease 1; TRY1; Cationic Trypsinogen; Protease Serine 1; Anionic Trypsin I; Pretrypsinogen I; Beta-Trypsin; EC 3.4.21.4; TRYP1; TRP1; Nonfunctional Trypsin 1; Digestive Zymogen; Anionic Trypsin-I; TCR V Beta 4.1; Trypsinogen 1; Trypsinogen A; Trypsin 1; Trypsin I; Trypsin-1; EC 3.4.21; TRY4 |
| *RAB24* | Q969Q5 | *RAB24*; RAB24; Member RAS Oncogene Family; Ras-Related Protein Rab-24 |
| *RHOT1* | Q8IXI2 | *RHOT1*; Ras Homolog Family Member T1; MIRO-1; MIRO1; ARHT1; Ras Homolog Gene Family Member T1; Mitochondrial Rho (MIRO) GTPase 1; Mitochondrial Rho GTPase 1; FLJ11040; Rac-GTP Binding Protein-Like Protein; Rac-GTP-Binding Protein-Like Protein; EC 3.6.5.-; EC 3.6.5; HMiro-1 |
| *SCGN* | O76038 | *SCGN*; secretagogin, EF-hand calcium binding protein; DJ501N12.8; SEGN; CALBL; calbindin-like; setagin; secretagogin |
| *SCPEP1* | Q9HB40 | *SCPEP1*; Serine Carboxypeptidase 1; RISC; Retinoid-Inducible Serine Carboxypeptidase; Serine Carboxypeptidase 1 Precursor Protein; Retinoid Inducible Serine Carboxypeptidase; EC 3.4.16.-; EC 3.4.16; HSCP1; SCP1 |
| *SERPINC1* | P01008 | *SERPINC1*; serpin family C member 1; ATIII; MGC22579; antithrombin III; signal peptide antithrombin part 1; coding sequence signal peptide antithrombin part 1; antithrombin (aa 375-432); serine (or cysteine) proteinase inhibitor, clade C (antithrombin), member 1; serpin peptidase inhibitor, clade C (antithrombin), member 1; AT3; AT3D; ATIII-R2; ATIII-T1; ATIII-T2; THPH7; serpin peptidase inhibitor clade C member 1; Antithrombin-III; Serpin C1 |
| *SERPINH1* | P50454 | *SERPINH1*; serpin family H member 1; HSP47; collagen binding protein 1; colligin; heat shock protein 47; serine (or cysteine) proteinase inhibitor, clade H (heat shock protein 47), member 2; serine (or cysteine) proteinase inhibitor, clade H (heat shock protein 47), member 1, (collagen binding protein 1); serpin peptidase inhibitor, clade H (heat shock protein 47), member 1, (collagen binding protein 1); AsTP3; CBP1; CBP2; OI10; PIG14; PPROM; RA-A47; SERPINH2; gp46; serpin H1; 47 kDa heat shock protein; arsenic-transactivated protein 3; cell proliferation-inducing gene 14 protein; colligin-1; colligin-2; rheumatoid arthritis antigen A-47; rheumatoid arthritis-related antigen RA-A47; serine (or cysteine) proteinase inhibitor, clade H (heat shock protein 47), member 2, (collagen-binding protein 2); Collagen-binding protein |
| *SFRP4* | Q6FHJ7 | *SFRP4*; secreted frizzled related protein 4; frpHE; FRP-4; FRZB-2; secreted frizzled-related protein 4; PYL; sFRP-4; frizzled protein, human endometrium; secreted frizzled-related protein 4; secreted frizzled-related protein 4 |
| *SHMT1* | P34896 | *SHMT1*; Serine Hydroxymethyltransferase 1; SHMT; CSHMT; Cytoplasmic Serine Hydroxymethyltransferase; Serine Hydroxymethyltransferase 1 (Soluble); Serine Hydroxymethyltransferase Cytosolic; Glycine Hydroxymethyltransferase; Serine Methylase; EC 2.1.2.1; MGC15229; MGC24556; 14 KDa Protein |
| *SMAD1* | Q15797 | *SMAD1*; SMAD family member 1; MADR1; JV4-1; MAD, mothers against decapentaplegic homolog 1 (Drosophila); SMAD, mothers against DPP homolog 1 (Drosophila); BSP-1; BSP1; JV41; MADH1; mothers against decapentaplegic homolog 1; MAD, mothers against decapentaplegic homolog 1; Mad-related protein 1; SMAD, mothers against DPP homolog 1; TGF-beta signaling protein 1; mothers against DPP homolog 1; transforming growth factor-beta signaling protein 1; MAD homolog 1; SMAD 1; hSMAD1; Transforming growth factor-beta-signaling protein 1 |
| *SOCS3* | O14543 | *SOCS3*; Suppressor Of Cytokine Signaling 3; SOCS-3; SSI-3; CIS3; Cytokine-Inducible SH2 Protein 3; STAT-Induced STAT Inhibitor 3; Cish3; SSI3; ATOD4; CISH3; CIS-3 |
| *SPART* | Q8N0X7 | *SPART*; spartin; KIAA0610; TAHCCP1; spastic paraplegia 20 (Troyer syndrome); SPG20; trans-activated by hepatitis C virus core protein 1; Spastic paraplegia 20 protein |
| *SPATA22* | Q8NHS9 | *SPATA22*; Spermatogenesis Associated 22; NYD-SP20; Spermatogenesis-Associated Protein 22; Testis Development Protein NYD-SP20; Testicular Tissue Protein Li 186; NYDSP20 |
| *STAT1* | P42224 | *STAT1*; signal transducer and activator of transcription 1; STAT91; ISGF-3; transcription factor ISGF-3 components p91/p84; signal transducer and activator of transcription 1, 91kD; signal transducer and activator of transcription 1, 91kDa; CANDF7; IMD31A; IMD31B; IMD31C; signal transducer and activator of transcription 1-alpha/beta |
| *STAT3* | P40763 | *STAT3*; signal transducer and activator of transcription 3; APRF; signal transducer and activator of transcription 3 (acute-phase response factor); ADMIO; ADMIO1; HIES; DNA-binding protein APRF; acute-phase response factor |
| *SYK* | P43405 | *SYK*; spleen associated tyrosine kinase; spleen tyrosine kinase; IMD82; p72-Syk; tyrosine-protein kinase SYK |
| *TBC1D13* | Q9NVG8 | *TBC1D13*; TBC1 Domain Family Member 13; FLJ10743; Epididymis Secretory Sperm Binding Protein |
| *TF* | P02787 | *TF*; transferrin; PRO1557; PRO2086; serotransferrin; HEL-S-71p; TFQTL1; beta-1 metal-binding globulin; epididymis secretory sperm binding protein Li 71p; siderophilin |
| *USP8* | P40818 | *USP8*; ubiquitin specific peptidase 8; HumORF8; KIAA0055; UBPY; SPG59; Ubiquitin carboxyl-terminal hydrolase 8; Ubiquitin isopeptidase Y; ubiquitin specific protease 8; PITA4; deubiquitinating enzyme 8; ubiquitin thiolesterase 8; ubiquitin-specific-processing protease 8; hUBPy; Ubiquitin thioesterase 8 |
| *WFIKKN2* | Q8TEU8 | *WFIKKN2*; WAP, follistatin/kazal, immunoglobulin, kunitz and netrin domain containing 2; WFIKKNRP; WFDC20B; WAP four-disulfide core domain 20B; GASP-1; hGASP-1; WAP, Kazal, immunoglobulin, Kunitz and NTR domain-containing protein 2; WAP, FS, Ig, two KU and NTR module related protein; WAP, follistatin, immunoglobulin, kunitz and NTR domain-containing-related protein; WFIKKN-related protein; growth and differentiation factor-associated serum protein 1; multivalent protease inhibitor protein; GASP1 |
| *YWHAB* | P31946 | *YWHAB*; Tyrosine 3-Monooxygenase/Tryptophan 5-Monooxygenase Activation Protein Beta; Tyrosine 3-Monooxygenase/Tryptophan 5-Monooxygenase Activation Protein Alpha Polypeptide; Tyrosine 3-Monooxygenase/Tryptophan 5-Monooxygenase Activation Protein Beta Polypeptide; 14-3-3 Protein Beta/Alpha; 14-3-3 Alpha; Protein 1054; KCIP-1; YWHAA; Protein Kinase C Inhibitor Protein-1; Protein Kinase C Inhibitor Protein 1; Epididymis Secretory Protein Li 1; 14-3-3 Beta; HEL-S-1; GW128; HS1 |
| *YWHAZ* | P63104 | *YWHAZ*; tyrosine 3-monooxygenase/tryptophan 5-monooxygenase activation protein zeta; KCIP-1; 14-3-3-zeta; 14-3-3 zeta; 14-3-3 delta; tyrosine 3-monooxygenase/tryptophan 5-monooxygenase activation protein, delta polypeptide; tyrosine 3-monooxygenase/tryptophan 5-monooxygenase activation protein, zeta polypeptide; HEL-S-3; HEL-S-93; HEL4; POPCHAS; YWHAD; 14-3-3 protein zeta/delta; 14-3-3 protein/cytosolic phospholipase A2; epididymis luminal protein 4; epididymis secretory protein Li 3; epididymis secretory protein Li 93; phospholipase A2; protein kinase C inhibitor protein-1; tyrosine 3/tryptophan 5 -monooxygenase activation protein, zeta polypeptide; Protein kinase C inhibitor protein 1 |

**S6 Table. Genes referenced in the manuscript and their aliases**. This table lists all gene names referenced in the manuscript, their UniProt identifiers, protein names, and gene aliases.
