# Supplementary material for: Identification of novel plasma proteomic biomarkers of Dupuytren disease
Source: PLoS One. 2026 Mar 18;21(3):e0343733. doi: 10.1371/journal.pone.0343733 (PMC12998848; doi:10.1371/journal.pone.0343733)
Supplement: S7 Table — Six proteins identified by Mass Spectrometry had statistically significant differences between Dupuytren and control cohorts in the SomaScan analysis, representing genes AOC3, POSTN, SERPINC1, TF, PCSK9, and KNG1. Within this network, AOC3, POSTN, and PCSK9 had strong pathway connections, and PCSK9, SERPINC1, TF, and KNG1 had functionally enriched pathways. PPI enrichment p-value: 0.00987. STR: Strength of association. FDR: False Discovery Rate (adjusted p-value). (DOCX) [file pone.0343733.s013.docx]

| **Category** | **ID** | **Description** | **STR** | **FDR** | **Matching proteins in this network** |
| --- | --- | --- | --- | --- | --- |
| Reactome | HSA-381426 | Regulation of Insulin-like Growth Factor (IGF) transport and uptake by Insulin-like Growth Factor Binding Proteins (IGFBPs) | 2.02 | 3.24E-05 | PCSK9, SERPINC1, TF, KNG1 |
| Reactome | HSA-8957275 | Post-translational protein phosphorylation | 2.09 | 3.24E-05 | PCSK9, SERPINC1, TF, KNG1 |

**S7 Table. Enriched pathways in Mass Spec - SomaScan concordant genes**. Six proteins identified by Mass Spectrometry had statistically significant differences between Dupuytren and control cohorts in the SomaScan analysis, representing genes AOC3, POSTN, SERPINC1, TF, PCSK9, and KNG1. Within this network, AOC3, POSTN, and PCSK9 had strong pathway connections, and PCSK9, SERPINC1, TF, and KNG1 had functionally enriched pathways. PPI enrichment p-value: 0.00987. STR: Strength of association. FDR: False Discovery Rate (adjusted p-value).
